# A year in pharmacology: new drugs approved by the US Food and Drug Administration in 2023

**DOI:** 10.1007/s00210-024-03063-1

**Published:** 2024-03-26

**Authors:** Gizem Kayki-Mutlu, Zinnet Sevval Aksoyalp, Leszek Wojnowski, Martin C. Michel

**Affiliations:** 1 Department of Pharmacology, Faculty of Pharmacy, Ankara University, Ankara, Türkiye; 2 Department of Pharmacology, Faculty of Pharmacy, Izmir Katip Celebi University, Izmir, Türkiye; 3 Department of Pharmacology, University Medical Center, Johannes Gutenberg University, Langenbeckstr. 1, 55118 Mainz, Germany

**Keywords:** FDA, New drugs, First-in-class, Next-in-class

## Abstract

With 54 new drugs and seven cellular and gene therapy products, the approvals by the US Food and Drug Administration (FDA) recovered 2023 from the 2022 dent back to the levels of 2020–2021. As in previous years of this annual review, we assign these new drugs to one of three levels of innovation: first drug against a condition (“first-in-indication”), first drug using a novel molecular mechanism (“first-in-class”), and “next-in-class,” i.e., a drug using an already exploited molecular mechanism. We identify four (7%) “first-in-indication,” 22 (36%) “first-in-class,” and 35 (57%) “next-in-class” drugs. By treatment area, rare diseases (54%) and cancer drugs (23%) were once again the most prevalent (and partly overlapping) therapeutic areas. Other continuing trends were the use of accelerated regulatory approval pathways and the reliance on biopharmaceuticals (biologics). 2023 marks the approval of a first therapy based on CRISPR/Cas9 gene editing.

## Introduction

Analyzing patterns of new drug approval testifies to the activities and priorities of the pharmaceutical industry and provides information on trends in novel treatment approaches. The US Food and Drug Administration (FDA) approved 37 new molecular entities in 2022 (Kayki-Mutlu et al. 2023), 50 in 2021 (Kayki-Mutlu et al. 2022), and 53 in 2020 (Kayki-Mutlu and Michel 2021). The dip in new approvals in 2022 apparently reflected reduced trial completions and regulatory filings during the COVID-19 pandemic. With 54 drugs plus seven cellular and gene therapy products in 2023, the approvals have recovered to the levels in 2020 and 2021. As in our previous annual reviews, we briefly summarize key efficacy and tolerability data. We classify the degree of innovation as first-in-indication, i.e., drugs for the treatment of a condition for which no approved medical treatments existed; first-in-class, i.e., drugs with a molecular mechanism of action that had not been used by previously approved medical treatments; and next-in-class, i.e., novel chemical or biological entities that exploit a molecular mechanism already available for the treatment of the same condition (Table 1). Table 2 breaks down the approvals according to the molecular structure (small molecule, antibody, peptide and protein, cellular and gene therapy). The increasingly common orphan drug status is given in Table 3. All approvals are discussed according to therapeutic areas.

**Table 1:** Newly approved drugs grouped by novelty. For definitions, see Introduction. Percentages are those of first-in-indication, first-in-class, and next-in-class drugs with all drugs (included cellular and gene therapies) approved in 2023 taken as 100%. Where available, the International Nonproprietary Name stems in drug names have been highlighted by underlining based on the WHO Stem Book (https://cdn.who.int/media/docs/default-source/international-nonproprietary-names-(inn)/inn-bio-review-2022.pdf?sfvrsn=f8db166f_3&download=true; https://cdn.who.int/media/docs/default-source/international-nonproprietary-names-(inn)/stembook-2018.pdf)

First-in-indication (*n* = 4, 7%)	Approved for	First-in-class (*n* = 22, 36%)	Approved for	Next-in-class (*n* = 35, 57%)	Approved for
Leniolisib	Phosphoinositide 3-kinase delta syndrome	Beremagene geperpavec-svdt	Dystrophic epidermolysis bullosa	Avacincaptad pegol	Geographic atrophy
Omaveloxolone	Friedreich’s ataxia	Birch triterpenes	Dystrophic and junctional epidermolysis	Bexagliflozin	Type 2 diabetes mellitus
Palovarotene	Heterotopic ossification	Capivasertib	Breast cancer	Bimekizumab-bkzx	Psoriasis
Pozelimab-bbfg	CHAPLE Disease	Daprodustat	Anemia	Cipaglucosidase Alfa-Atga	Late-onset Pompe disease
		Delandistrogene moxeparvovec-rokl	Duchenne muscular dystrophy	Efbemalenograstim alfa-vuxw	Neutropenia
		Donislecel-jujn	Type 1 diabetes mellitus	Elacestrant	Breast cancer
		Exagamglogene autotemcel	Sickle cell disease	Elranatamab-bcmm	Relapsed or refractory multiple myeloma
		Fezolinetant	Menopause	Epcoritamab-bysp	Diffuse large B cell lymphoma
		Iptacopan	Paroxysmal nocturnal hemoglobinuria	Eplontersen	Polyneuropathy of hereditary transthyretin-mediated amyloidosis
		Lotilaner	Demodex blepharitis	Etrasimod	Ulcerative colitis
		Lovotibeglogene autotemcel	Sickle cell disease	Flotufolastat F 18	Prostate-specific membrane antigen (PSMA)-positive lesions
		Nedosiran	Primary hyperoxaluria	Fruquintinib	Metastatic colorectal cancer
		Nirmatrelvir, ritonavir	COVID-19	Gepirone	Major depressive disorder
		Nirogacestat	Progressing desmoid tumors	Glofitamab-gxbm	Diffuse large B cell lymphoma
		Omidubicel-onlv	Hematologic malignancies	Lecanemab-irmb	Alzheimer’s disease
		Perfluorohexyloctane	Dry eye disease	Mirikizumab-mrkz	Ulcerative colitis
		Sparsentan	Proteinuria in primary immunoglobulin A nephropathy	Momelotinib	Myelofibrosis
		Talquetamab-tgvs	Relapsed or refractory multiple myeloma	Motixafortide	Multiple myeloma
		Tofersen	Amyotrophic lateral sclerosis	Nirsevimab-alip	RSV
		Trofinetide	Rett syndrome	Pegunigalsidase Alfa-Iwxj	Alpha-galactosidase A deficiency
		Valoctocogene roxaparvovec-rvox	Hemophilia A	Pirtobrutinib	Mantle cell lymphoma
		Velmanase alfa-tycv	Alpha-mannosidase	Quizartinib	Acute myeloid leukemia
				Repotrectinib	ROS1-positive non-small cell lung cancer
				Retifanlimab-dlwr	Merkel cell carcinoma
				Rezafungin	Candidiasis
				Ritlecitinib	Alopecia areata
				Rozanolixizumab-noli	Myasthenia gravis
				Somatrogon-ghla	Growth hormone deficiency
				Sotagliflozin	Heart failure
				Sulbactam, durlobactam	Pneumonia
				Toripalimab-tpzi	Metastatic or recurrent nasopharyngeal carcinoma
				Vamorolone	Duchenne muscular dystrophy
				Zavegepant	Migraine
				Zilucoplan	Myasthenia gravis
				Zuranolone	Postpartum depression

**Table 2:** Newly approved drugs grouped by drug type. Percentages are those of small molecule, antibody, peptide-protein drugs, cellular and gene therapy, with all drugs approved by the FDA in 2023 taken as 100%

Small molecule (*n* = 30, 49%)	Antibody (*n* = 12, 20%)	Peptide and protein (*n* = 8, 13%)	Nucleic-acid based (*n* = 4, 7%)	Cellular and gene products (*n* = 7, 11%)
Bexagliflozin	Bimekizumab-bkzx	Cipaglucosidase alfa-atga	Avacincapted pegol	Beremagene geperpavec-svdt
Birch triterpenes	Elranatamab-bcmm	Efbemalenograstim alfa-vuxw	Eplontersen	Delandistrogene moxeparvovec-rokl
Capivasertib	Epcoritamab-bysp	Motixafortide	Nedosiran	Donislecel-jujn
Daprodustat	Glofitamab-gxbm	Pegunigalsidase alfa-iwxj	Tofersen	Exagamglogene autotemcel
Elacestrant	Lecanemab-irmb	Somatrogon-ghla		Lovotibeglogene autotemcel
Etrasimod	Mirikizumab-mrkz	Trofinetide		Omidubicel-onlv
Fezolinetant	Nirsevimab-alip	Velmanase alfa-tycv		Valoctocogene roxaparvovec
Flotufolastat F 18	Pozelimab-bbfg	Zilucoplan		
Fruquintinib	Retifanlimab-dlwr			
Gepirone	Rozanolixizumab-noli			
Iptacopan	Talquetamab-tgvs			
Leniolisib	Toripalimab-tpzi			
Lotilaner				
Momelotinib				
Nirmatrelvir, ritonavir				
Nirogacestat				
Omaveloxolone				
Palovarotene				
Perfluorohexyloctane				
Pirtobrutinib				
Quizartinib				
Repotrectinib				
Rezafungin				
Ritlecitinib				
Sotagliflozin				
Sparsentan				
Sulbactam, durlobactam				
Vamorolone				
Zavegepant				
Zuranolone

**Table 3:** 2023 FDA orphan drug approvals. Percentage is that of orphan drugs within all drugs approved by the FDA in 2023 taken as 100% (https://www.accessdata.fda.gov/scripts/opdlisting/oopd/listResult.cfm)

Drug (*n* = 33, 54%)	Major indications
Beremagene geperpavec-svdt	Dystrophic epidermolysis bullosa
Birch Triterpenes	Dystrophic and junctional epidermolysis
Cipaglucosidase alfa-atga	Late-onset Pompe disease
Elranatamab-bcmm	Relapsed or refractory multiple myeloma
Eplontersen	Transthyretin-mediated amyloidosis
Exagamglogene autotemcel	Sickle cell disease
Iptacopan	Paroxysmal nocturnal hemoglobinuria
Leniosilib	Phosphoinositide 3-kinase delta syndrome
Lovotibeglogene autotemcel	Sickle cell disease
Momelotinib	Myelofibrosis
Motixafortide	Stem cell mobilization
Nedosiran	Primary hyperoxaluria
Nirogacestat	Desmoid tumors
Omaveloxolone	Friedreich’s ataxia
Omidubicel-onlv	Hematologic malignancies
Palovarotene	Heterotopic ossification
Pirtobrutinib	Mantle cell lymphoma
Pozelimab-bbfg	CHAPLE disease
Quizartinib	Acute myeloid leukemia
Repotrectinib	ROS1-positive non-small cell lung cancer
Retifanlimab-dlwr	Merkel cell carcinoma
Rezafungin	Candidiasis
Rozanolixizumab-noli	Myasthenia gravis
Somatrogon-ghla	Growth hormone deficiency
Sparsentan	Proteinuria in primary immunoglobulin A nephropathy
Talquetamab-tgvs	Relapsed or refractory multiple myeloma
Tofersen	Amyotrophic lateral sclerosis
Toripalimab-tpzi	Metastatic or recurrent nasopharyngeal carcinoma
Trofinetide	Rett syndrome
Valoctocogene roxaparvovec	Hemophilia A
Vamorolone	Duchenne muscular dystrophy
Velmanase alfa-tycv	Alpha-Mannosidase
Zilucoplan	Myasthenia gravis

It is explicitly not our intention to compare novel treatments with their specific advantages and disadvantages with existing ones, because this is best done by experts in a therapeutic area. Similarly, we do not discuss drug pricing for novel treatments. Such discussion can only be meaningful based on input from experts within a specific therapeutic area who can judge on the added clinical value of a treatment. They fall into the responsibility of Health Technology Assessment bodies such as the National Institute for Health and Care Excellence in the UK.

## Methods

Our analyses follow the same protocol as those for newly approved drugs in 2020–2022 (Kayki-Mutlu and Michel 2021; Kayki-Mutlu et al. 2022, 2023) with one exception. Unlike in previous years, we have included cellular and gene therapies approved by the FDA (U.S. Food and Drug Administration 2023a, b) into our analysis. However, we exclude generics, or generic versions of biopharmaceuticals (“biosimilars”), and already approved drugs that received marketing authorizations for one or more additional indications and/or in a novel formulation in 2023. Newly approved drug combinations were only considered if at least one of the combination partners (mostly therapeutic antibodies) is a novel chemical or biopharmaceutical entity. We would like to emphasize that other regulatory agencies may have approved the same compounds earlier than the FDA (among this year’s approvals, e.g., toripalimab and fruquintinib in China and daprodustat in Japan), may do so at later points in time, may choose not to approve some of these compounds, or may choose to approve compounds not approved by the FDA. Our focus on drug approvals by the FDA does not imply any opinion on the scientific quality of approvals by the FDA as compared to the regulatory authorities in other jurisdictions, but rather uses the FDA as a point of reference, due to its status as one of the most influential drug regulatory authorities. All indications refer to adults unless specifically noted otherwise.

## Oncology

Bruton tyrosine kinase (BTK) is involved in B cell development and maturation and plays a role in B cell malignancies such as mantle cell lymphoma (Keam 2023c). BTK inhibitors are the standard treatment for B cell malignancies (Hatashima et al. 2022). The C481S mutation of BTK is the most common resistance mechanism restricting the treatment efficacy of covalent BTK inhibitors (Naeem et al. 2023). The next-in-class small molecule *pirtobrutinib* is an oral, potent, highly selective, and noncovalent BTK inhibitor regardless of the BTK C481S mutation status (Mato et al. 2021). Pirtobrutinib was first approved in January 2023 under accelerated approval for treating of relapsed or refractory mantle cell lymphoma in adults following two or more lines of systemic therapy (Keam 2023c). In December 2023, pirtobrutinib was approved for chronic lymphocytic leukemia and small lymphocytic lymphoma. Pirtobrutinib received priority review, fast track designation, and orphan drug designation. Low levels of neutrophils, lymphocytes, platelets and hemoglobin, tiredness, pain, bruising, and diarrhea are the most frequent adverse effects (AE) of pirtobrutinib (Keam 2023c).

Diffuse large B cell lymphoma is the most common type of non-Hodgkin’s lymphoma and often relapses or is refractory to treatment, which limits the available treatment options (Riaz et al. 2023). Bispecific T cell recruiting antibodies are a class of immunotherapeutics used in treating B cell lymphomas that target both T-cells and antigens on malignant cells (Minson and Dickinson 2021). *Epcoritamab-bysp* is an immunoglobulin G (IgG)1-based bispecific antibody and CD20-directed CD3 T cell engager that binds to CD3 on T cells and CD20 on B cells (Engelberts et al. 2020). Epcoritamab has been approved for relapsed or refractory diffuse large B cell lymphoma and high-grade B cell lymphoma after at least two prior systemic treatments via priority review and accelerated approval (Frampton 2023). The most common treatment-related AE include cytokine release syndrome, injection area reactions, decreased neutrophile levels, tiredness, and pyrexia (Riaz et al. 2023).

Another CD20 x CD3 T cell-binding bispecific monoclonal antibody approved in 2023 is *glofitamab-gxbm* (Shirley 2023a). Glofitamab has been approved for relapsed or refractory diffuse, large B cell lymphoma, not otherwise specified, or large B cell lymphoma arising from follicular lymphoma after at least two prior systemic therapies. Glofitamab-gxbm received priority review and fast track designation. As with epcoritamab, the mechanism of action of glofitamab is T cell-mediated lysis of CD20-expressing B cells (Shirley 2023a). Glofitamab has a boxed warning for the risk of cytokine release syndrome. Other AE include neutropenia, anemia, and thrombocytopenia (Shirley 2023a).

G protein-coupled receptor family C group 5 member D (GPRC5D) shows high and selective expression on multiple myeloma cells and is a target for immunotherapeutic treatments (Verkleij et al. 2021). *Talquetamab-tgvs* is a humanized, bispecific GPRC5D-targeting T cell engager (Verkleij et al. 2021), which acts through binding to GPRC5D-expressing cells and CD3 on T cells (Kaplon et al. 2023). Talquetamab is the first IgG4 bispecific antibody treatment to be approved for the treatment of relapsed or refractory multiple myeloma in adults who have received at least four therapies (Keam 2023d). Talquetamab received priority review, breakthrough, and orphan drug designation, and underwent accelerated approval (Kaplon et al. 2023; Keam 2023d). The most frequent AE of talquetamab are fever, cytokine release syndrome, pain, tiredness, weight loss, xerostomia, dysphagia, upper respiratory tract infections, diarrhea, hypotension, taste, swallowing, and nail, and skin problems (Keam 2023d).

Another drug approved in 2023 via accelerated approval for treating relapsed or refractory multiple myeloma is *elranatamab-bcmm* (Dhillon 2023b). Elranatamab is a bispecific B cell maturation antigen (BCMA) directed-CD3^+^ T cell engager antibody (Rais et al. 2023). Elranatamab binds CD3 on T cell to BCMA on the surface of myeloma cells and induces a potent cytotoxic response against BCMA-expressing myeloma cells by activating T cells (Dhillon 2023b; Rais et al. 2023). Elranatamab has been approved for relapsed or refractory multiple myeloma after four or more prior therapies following priority review, breakthrough, and orphan drug designation (Dhillon 2023b). Elranatamab is well tolerated, and the most common non-hematologic treatment-emergent AE are cytokine release syndrome, diarrhea, tiredness, loss of appetite, and fever (Lesokhin et al. 2023).

Myelofibrosis is a progressive, Philadelphia chromosome-negative myeloproliferative neoplasm that is characterized by a dysregulation of the Janus kinase and signal transducer and activator of transcription (JAK-STAT) signaling pathway (Passamonti and Mora 2023). This leads to inflammation and fibrosis and to hyperactivation of the activin A receptor type I (ACVR1), which results in increased expression of hepcidin and impaired iron metabolism (Chifotides et al. 2022; Verstovsek et al. 2023). *Momelotinib* is a small molecule, first-in-class oral inhibitor of JAK1/JAK2 and ACVR1 (Keam 2023b; Verstovsek et al. 2023), which reduces the hepcidin expression and increases the availability of iron for erythropoiesis (Tremblay and Mesa 2022). Momelotinib is approved for intermediate or high-risk primary myelofibrosis and secondary myelofibrosis with anemia (Keam 2023b). Momelotinib was granted fast track and orphan drug designation. The most frequent AE are thrombocytopenia, diarrhea, bleeding, tiredness, nausea, infections, pain, itching, increased liver enzymes, and fever (Keam 2023b).

Recurrent or metastatic nasopharyngeal carcinoma is a rare malignancy. The standard first-line treatment is platinum-based chemotherapy, but survival outcomes are poor (Han et al. 2023). *Toripalimab-tpzi* is a selective, recombinant, programmed death receptor-1 (PD-1) monoclonal antibody that prevents the binding of programmed death ligands 1 (PD-L1) and 2 (PD-L2) to PD-1 receptors and improves the anti-tumor response of T cells (Zhang et al. 2021). Toripalimab was approved in China in 2018 to treat nasopharyngeal and urothelial carcinoma and in 2021 to treat unresectable or metastatic melanoma (Keam 2019; Zhang et al. 2021). In 2023, the FDA approved toripalimab for adults with metastatic or recurrent, locally advanced nasopharyngeal carcinoma, both as first-line therapy in combination with cisplatin and gemcitabine, and as monotherapy during or after platinum-containing chemotherapy. Toripalimab received priority review, breakthrough designation, and orphan drug designation. The most common AE of monotherapy are tiredness, hypothyroidism, and pain.

The standard treatment for metastatic colorectal cancer includes chemotherapy doublets/triplets combined with monoclonal antibodies against the epidermal growth factor receptor or the vascular endothelial growth factor receptor (VEGFR) (Lavacchi et al. 2023). *Fruquintinib* is a potent and highly selective tyrosine kinase inhibitor antibody against VEGFR1, 2, and 3 that inhibits angiogenesis (Shirley 2018). In 2018, fruquintinib was approved in China to treat metastatic colorectal cancer (Shirley 2018). In 2023, it was approved by the FDA via priority review for metastatic colorectal cancer in adults. The most common AE were increased blood pressure and thyroid-stimulating hormone levels, palmar-plantar erythrodysesthesia, proteinuria, and hoarseness (Shirley 2018).

A rearrangement of the ROS proto-oncogene 1 (ROS1) has been identified as a novel molecular subtype of non-small cell lung cancer (NSCLC), as ROS1 rearrangements enhance cell proliferation and decrease apoptosis (Kim et al. 2013). ROS1 fusion is rare (prevalence approximately 1–3% among NSCLC), but it is considered an interesting target for treating metastatic NSCLC (Dyrbekk et al. 2023). The development of resistance to ROS1 tyrosine kinase inhibitors over time requires novel inhibitors (Yun et al. 2020). The tyrosine kinase inhibitor *repotrectinib* was approved for locally advanced or metastatic ROS1-positive NSCLC. Repotrectinib received priority review, breakthrough, and fast track designation. The most common AE are dizziness, taste disorder, peripheral neuropathy, constipation, dyspnea, ataxia, tiredness, cognitive disorders, and muscular weakness.

Endocrine therapy plays a key role in the treatment of estrogen receptor (ER)-positive breast cancer. Mutations in the ESR1 gene encoding the ER-α receptor lead to the development of resistance and disease progression in ER-positive metastatic breast cancer patients receiving endocrine therapy (Lloyd et al. 2022). *Elacestrant* is a selective estrogen receptor degrader (Beumer and Foldi 2023). It is the first, oral, selective estrogen receptor degrader and has dose-dependent agonist/antagonist effects on the ER (Beumer and Foldi 2023). Elacestrant has been approved for treating postmenopausal women or adult men with ER-positive, human epidermal growth factor receptor 2 (HER2)-negative, ESR1 gene mutation-positive, advanced or metastatic breast cancer that has progressed after endocrine therapy (Hoy 2023b). Elacestrant received priority review and fast track designation. The safety profile of elacestrant as a monotherapy was manageable and acceptable in phase 1 and phase 3 trials (Bardia et al. 2021; Bidard et al. 2022). The most frequent AE were nausea and vomiting, tiredness, and loss of appetite (Hoy 2023b). Elacestrant is susceptible to drug interactions because it is mainly metabolized by CYP3A4 (Beumer and Foldi 2023).

Another drug approved by the FDA for breast cancer via priority review in 2023 is *capivasertib*. Capivasertib is a first-in-class, selective, highly potent ATP-competitive pan-AKT kinase (protein kinase B) inhibitor with similar activities against the three isoforms AKT1, AKT2, and AKT3 (Andrikopoulou et al. 2022; Mullard 2023). Capivasertib has been approved for treating hormone receptor-positive (estrogen or progesterone receptors, or both), HER2-negative, locally advanced or metastatic breast cancer in combination with the estrogen receptor antagonist fulvestrant in with the presence of one or more biomarker alterations (PIK3CA, AKT1 or PTEN) (Mullard 2023). The most frequent AE of the combination of capivasertib and fulvestrant are skin rash and diarrhea (Mullard 2023).

Merkel cell carcinoma is a rare, aggressive form of skin cancer. Anti-PD-1 and PD-L1 immunotherapy is recommended as a first-line systemic therapy for patients with advanced disease (Gauci et al. 2022). *Retifanlimab-dlwr* is a humanized monoclonal antibody that blocks PD-1, preventing PD-L1 binding and thereby stimulating an immune response against cancer by increasing T cell activity (Kang 2023). Retifanlimab was approved for metastatic or recurrent locally advanced Merkel cell carcinoma under accelerated approval, priority review, fast track, and orphan drug designation (Kang 2023). In addition, retifanlimab is in phase III trials for anal squamous cell carcinoma and NSCLC (Kang 2023). The most frequent AE are tiredness, pain, itching, diarrhea, rash, fever, and nausea (Kang 2023).

Activating mutations of the *fms*-like tyrosine kinase 3 (FLT3) gene are associated with a poor outcome in patients with acute myeloid leukemia (Ambinder and Levis 2021; Killock 2023). *Quizartinib*, a potent and specific oral FLT3 inhibitor, exerts an anti-cancer effect by inhibiting the activity of the mutation-activated FLT3, which causes tumor cells to undergo apoptosis (Naqvi and Ravandi 2019). Quizartinib has been approved for newly diagnosed acute myeloid leukemia that is FLT3 internal tandem duplication-positive. Quizartinib received priority review, fast track, and orphan drug designation. The safety profile of quizartinib is manageable (Kayser and Levis 2023).

Desmoid tumors, also known as aggressive fibromatosis, are rare, benign, invasive, non-metastatic, highly recurrent soft-tissue tumors (Gounder et al. 2023). *Nirogacestat*, an oral, selective, noncompetitive, reversible gamma-secretase inhibitor, has been approved for progressing desmoid tumors in adults who require systemic treatment (Zheng et al. 2024). Nirogacestat works by cleaving the Notch protein, which is part of the signaling pathway leading to the development of desmoid tumors (Rohail et al. 2023). Nirogacestat received priority review, fast track, breakthrough therapy, and orphan drug designation (Rohail et al. 2023). The most common adverse events are decreased phosphate levels and appetite, diarrhea, nausea-vomiting, tiredness, and skin rash (Messersmith et al. 2015).

## Hematology

Anemia is a common condition in people with chronic kidney disease. It is characterized by reduced red blood cell number due to insufficient erythropoietin production (Koury and Haase 2015). Hypoxia-inducible factors (HIF) mediate the cellular response to hypoxia, leading to an increase in the production of erythropoietin. HIF-specific prolyl hydroxylases induce HIF degradation (Sanghani and Haase 2019). Prolyl hydroxylase inhibitors to treat anemia associated with chronic renal failure are under development (Johansen et al. 2023). *Daprodustat*, a first-in-class reversible inhibitor of HIF-specific prolyl hydroxylase, was first approved in Japan for treating of renal anemia in 2020 (Dhillon 2020) by the FDA in 2023. Daprodustat is the first and only approved treatment for anemia due to chronic kidney disease in adults on dialysis for at least 4 months in the USA that is given orally (Haider et al. 2023). Serious AE did not differ between daprodustat and placebo groups in the phase III trial (Johansen et al. 2023).

Paroxysmal nocturnal hemoglobinuria is a non-malignant, rare blood disorder manifesting as hemolytic anemia, bone marrow failure, thrombosis, and complement deficiency (Brodsky 2014). *Iptacopan* is a first-in-class, oral, and selective inhibitor of complement factor B, which is an initiator in the complement alternative pathway (Han et al. 2022; Jang et al. 2022). In the complement alternative pathway, iptacopan controls both extravascular C3b-dependent and intravascular terminal complement-dependent hemolysis. It has been approved for paroxysmal nocturnal hemoglobinuria with breakthrough therapy and orphan drug designation. The most common AE are diarrhea, pain, infections, nausea, and skin rash.

Hemophilia A is an inherited bleeding disorder caused by a deficiency of the coagulation factor VIII which is an essential cofactor in the coagulation pathway (Fassel and McGuinn 2021). *Valoctocogene roxaparvovec-rvox* is an adeno-associated virus type-5 (AAV-5)-based gene therapy vector approved to treat severe hemophilia A in adults who do not have antibodies to AAV-5. Valoctocogene roxaparvovec was granted orphan drug, breakthrough therapy, and regenerative medicine advanced therapy designation (Blair 2022). The most frequent AE are raised liver enzymes, nausea, and pain (Blair 2022).

Febrile neutropenia is a type of neutropenia associated with high fever and increased susceptibility to infections in patients receiving myelosuppressive chemotherapy (Blayney and Schwartzberg 2022). Recombinant granulocyte-colony stimulating factor (G-CSF) is used to prevent neutropenia, but its short half-life of G-CSF limits its use. A long-acting G-CSF was developed using fusion protein technology (Blair 2023a). The first long-acting G-CSF product, eflapegrastim-xnst, was approved by the FDA last in 2022 (Kayki-Mutlu et al. 2023). In 2023, *efbemalenograstim alfa-vuxw* has been approved to reduce the incidence of infections with febrile neutropenia in adult patients with non-myeloid malignancies who are receiving myelosuppressive therapy and are at risk of developing febrile neutropenia. Efbemalenograstim alfa is a dimeric recombinant fusion protein, including two G-CSF molecules and an IgG2-Fc fragment (Blair 2023b). Efbemalenograstim alpha stimulates survival, proliferation, and differentiation of hematopoietic cells by specifically binding to the G-CSF receptor (Crawford and Oswalt 2023). The most common AE are nausea, anemia, and low platelet count.

Allogeneic hematopoietic cell transplantation is the sole potential curative therapy option in many hematological malignancies (Lin et al. 2023). Hematopoietic stem cells can be collected from umbilical cord blood as well as bone marrow and peripheral blood (Saiyin et al. 2023). The disadvantage of umbilical cord blood is that it provides low numbers of hematopoietic stem cells, which may lead to treatment failure. An ex vivo culture strategy has been developed to enhance the number of hematopoietic stem cells (Saiyin et al. 2023). *Omidubicel-onlv* is a nicotinamide-modified stem cell transplant that is derived from the blood of the umbilical cord (Heo 2023a). Omidubicel is the first-in-class allogeneic hematopoietic progenitor cell therapy approved for adults and children (≥ 12 years) with hematological malignancies undergoing cord blood transplantation after myeloablative conditioning to reduce the risk of infection and the time to neutrophil recovery (Heo 2023a). Omidubicel received priority review, breakthrough, and orphan drug designation. Infusion reactions, infections, and graft-versus-host disease have been reported as AE with omidubicel (Heo 2023a).

Sufficient hematopoietic stem cell mobilization during treatment is necessary for autologous stem cell transplantation to be successful in patients with multiple myeloma (Giralt et al. 2014). The interaction of the C-X-C motif chemokine ligand 12 (CXCL12) with its receptor CXCR4 enables hematopoietic stem cells to be retained in the bone marrow, and CXCL12/CXCR4 axis inhibition is a notable therapeutic approach (Ladikou et al. 2020). *Motixafortide* is a selective CXCR4 inhibitor that acts as a hematopoietic stem cell mobilizer (Crees et al. 2023a). Motixafortide was approved for use in combination with filgrastim to mobilize hematopoietic stem cells to the peripheral blood in patients with multiple myeloma (Hoy 2023d). Motixafortide was found to be well tolerated, with the most frequent AE being transient injection-site reactions (pain, erythema, and pruritis) and systemic reactions (flushing, pruritis, urticaria, and erythema) (Crees et al. 2023b).

## Neurology

In Alzheimer’s disease, the accumulation of amyloid-β aggregates is believed to be the trigger for the onset and progression of the pathological processes (Yadollahikhales and Rojas 2023). The first anti-amyloid-β antibody, aducanumab, received FDA approval in 2021 (Kayki-Mutlu et al. 2022). Anti-amyloid immunotherapy has emerged as the first effective disease-modifying treatment for Alzheimer’s disease (Yadollahikhales and Rojas 2023). *Lecanemab-irmb* is an IgG1 monoclonal antibody that binds aggregated soluble and insoluble forms of amyloid-β peptide (Hoy 2023c). Lecanemab-irmb received fast track, priority review, and breakthrough therapy designations and received accelerated approval. The presence of amyloid-ß pathology should be confirmed prior to the initiation of lecanemab, and treatment should be initiated in patients with a mild stage of the disease (Hoy 2023c). The most frequent AE with lecanemab are infusion-related reactions, amyloid-related imaging abnormalities, headache, and falls (Hoy 2023c). The risk of amyloid-related imaging abnormalities (brain swelling or bleeding) is a safety concern, particularly in patients taking anticoagulants (Couzin-Frankel and Piller 2022).

Friedreich’s ataxia is a rare, progressive genetic neurodegenerative disease and involves a defect in the *nuclear factor (erythroid-derived 2)-like 2 (Nrf2)* antioxidant signaling pathway (Abeti et al. 2018). Suppression of the *Nrf2* signaling pathway leads to oxidative stress, mitochondrial dysfunction, and cellular damage (Abeti et al. 2018). *Omaveloxolone*, an oral *Nrf2* inducer, enhances the antioxidant activity of the *Nrf2* pathway (Abeti et al. 2018). Omaveloxolone has been approved as first-in-indication treatment of Friedreich’s ataxia in people older than 16 years (Lee 2023). Omaveloxolone has been granted orphan drug, fast track, priority review, and rare pediatric disease designations (Dayalan Naidu and Dinkova-Kostova 2023). Frequent AE include increased liver enzymes, headache, nausea, pain, tiredness, and diarrhea (Lee 2023).

Two drugs gained approval for the treatment of Duchenne muscular dystrophy (DMD), a rare X-linked genetic neuromuscular disorder resulting from mutations in the DMD gene, which encodes dystrophin, a protein crucial for proper muscle function (Keam 2023f). One of these drugs is *vamorolone*, a synthetic glucocorticoid designed to exert anti-inflammatory and immunosuppressant effects in patients over 2 years old with DMD. Orally administered vamorolone has demonstrated effectiveness in improving various parameters over a 24-week period in patients, including the time to stand from supine, 6-min walk distance, time to run/walk 10 m, and time to climb 4 stairs. Additionally, bone turnover markers were found to be reduced with prednisone but not with vamorolone treatment (Guglieri et al. 2022). Long-term vamorolone treatment has been reported to exhibit reduced side effects compared to traditional glucocorticoid treatment (Mah et al. 2022). Reported side effects include Cushingoid features, psychiatric problems, vomiting, increased weight, and vitamin D deficiency. Vamorolone has received designations for fast track, orphan drug, and rare pediatric disease.

The second drug approved for treating DMD marks a groundbreaking advancement as the first gene therapy indicated for this disease. *Delandistrogene moxeparvovec-rokl* is a non-replicating, recombinant adeno-associated virus vector containing a microdystrophin transgene, which confers dystrophin activity. This therapy is specifically intended for use in children with a confirmed DMD gene mutation, who can still walk, and is administered as a single-dose intravenous infusion. Results from clinical studies have confirmed the achievement of dystrophin expression with delandistrogene therapy, leading to improved motor functions as assessed by North Star Ambulatory Assessment scores. Notably, the medication has demonstrated a high level of tolerability (Mendell et al. 2023; Zaidman et al. 2023). The most commonly reported AE include vomiting and nausea, pyrexia, and thrombocytopenia. The therapy has been granted accelerated approval.

The release of calcitonin gene-related peptide (CGRP) is involved in the migraine pathophysiology and monoclonal antibodies and small molecule CGRP receptor antagonists have been approved against this disease (Nisar et al. 2023). Zavegepant is a third generation, highly potent, selective, competitive, intranasally administered CGRP receptor antagonist that is approved for the acute, but not for the prophylactic treatment, of migraine (Dhillon 2023c; Larik et al. 2023). Intranasal zavegepant may benefit patients with migraine for whom oral medications are ineffective, slow-acting, or intolerable due to vomiting (Lipton et al. 2023). The most frequent AE are taste disorders, nausea-vomiting, and nasal discomfort (Dhillon 2023c).

Rett syndrome is a rare genetic neurodevelopmental disorder that is characterized by X-linked *methyl-CpG-binding protein 2* (*MECP2*) mutation that can lead to permanent neurological damage or death in children (Hudu et al. 2023; Parent et al. 2023). *Trofinetide* is the first treatment approved for Rett syndrome in adults and pediatric patients (≥ 2 years) (Keam 2023e). Trofinetide was granted priority review, orphan, and fast track drug designations. Trofinetide is a synthetic peptidase-resistant analogue of the neuroprotective tripeptide glycine-proline-glutamate, a product of the cleavage of insulin-like growth factor 1 in the brain (Hudu et al. 2023). The exact mechanism of action of trofinetide is not fully understood. However, there is evidence that it modulates the insulin-like growth factor 1 pathway, improves synaptic function, and reduces levels of inflammatory mediators, apoptosis, and oxidation (Hudu et al. 2023; Parent et al. 2023). The most frequent AE are diarrhea and vomiting (Keam 2023e).

Amyotrophic lateral sclerosis (ALS) is a neurodegenerative disease, and a mutation in the superoxide dismutase 1 (SOD1) gene is the cause of about 2% of adult cases of ALS (Jin and Zhong 2023; Mead et al. 2023). The development of disease-modifying gene therapies is a priority in ALS. *Tofersen* is an intrathecally administered antisense oligonucleotide and is the first approved treatment for ALS in adults with a confirmed mutation in the SOD1 gene (Blair 2023c). Tofersen was granted priority review and orphan drug and fast track drug designations. Tofersen received an accelerated approval because it was shown to reduce plasma neurofilament light chain, a biomarker of neurodegeneration (Blair 2023c; Jin and Zhong 2023). The mechanism of action of tofersen involves binding to the SOD1 mRNA, leading to decreased synthesis of the SOD1 protein (Mead et al. 2023). The most frequent AE are pain, tiredness, and increased white blood cells in the cerebrospinal fluid (Blair 2023c).

Myasthenia gravis is a chronic, neuroimmunological disorder attributed to autoantibodies affecting post-synaptic neuromuscular junctions in the skeletal muscle (Menon and Bril 2022). In most cases of myasthenia gravis, IgG antibodies block the binding of acetylcholine to the nicotinic acetylcholine receptor (AChR) and muscle-specific kinase (MuSK), and AChR antibodies induce internalization of the AChR and destruction of synaptic transmission at the neuromuscular junction (Menon and Bril 2022). *Rozanolixizumab-noli* is an IgG4 monoclonal antibody that blocks the human neonatal Fc receptor and inhibits the interaction of the Fc receptor with IgG4, resulting in the removal of unbound IgG4 via the lysosomal degradation pathway (Smith et al. 2018; Menon and Bril 2022). Rozanolixizumab is the first drug approved to treat both anti-AChR and anti-MuSK antibody-positive adult generalized myasthenia gravis (Hoy 2023e). It received the priority review and orphan drug designation. The most frequent AE were headache, diarrhea, fever, and nausea (Hoy 2023e).

Another drug approved by the FDA in 2023 for treating generalized myasthenia gravis is *zilucoplan*. Zilucoplan is a small, synthetic macrocyclic peptide inhibitor of the complement component 5 (Shirley 2023b), by preventing the cleavage of C5 into C5a and C5b and also by inhibiting the binding of C5b to C6 (Tang et al. 2023). Thus, zilucoplan has been suggested to have a dual mechanism of action preventing complement activation (Tang et al. 2023). Zilucoplan has been approved for use in adult patients who are positive for anti-AChR antibodies (Shirley 2023b) and granted orphan drug status (Menon and Bril 2022). The most common AE are bruising and pain at the injection site, headache, diarrhea, and exacerbation of the disease (Shirley 2023b).

Postpartum depression is a major form of depression, and the effects of fluctuations in neurosteroid levels on the expression of gamma-aminobutyric acid (GABA)_A_ receptors are thought to play a role in its pathogenesis (Gunduz-Bruce et al. 2022). Therefore, GABA modulators have been investigated in the treatment of postpartum depression. *Zuranolone* is an oral, synthetic neuroactive steroid and a positive allosteric modulator of the GABA_A_ receptor (Heo 2023b). Zuranolone is the first therapy approved for the treatment of postpartum depression that is given orally (Heo 2023b). Zuranolone received priority review and fast track designation. The most common AE are drowsiness, headache, dizziness, sedation, nausea, urinary tract infections, fatigue, and diarrhea (Heo 2023b).

*Gepirone* has been in development for several decades against major depressive disorder (Jenkins et al. 1990). Gepirone is an oral, selective serotonin 5HT_1A_ receptor agonist, and its extended-release formulation is the first approval for the treatment of major depressive disorder in adults (Keam 2023a). Gepirone has shown good tolerability in clinical trials, with the most frequent AE being dizziness, pain, drowsiness, insomnia, gastrointestinal upset, upper respiratory tract infections, xerostomia, and enhanced appetite (Keam 2023a).

Hereditary transthyretin amyloidosis with polyneuropathy is a rare, progressive disease that causes damage to the peripheral nerves because of the accumulation of transthyretin amyloid fibrils (Adams et al. 2021). *Eplontersen* is an N-acetylgalactosamine-linked antisense oligonucleotide that inhibits transthyretin production and decreases the serum and tissue transthyretin levels (Coelho et al. 2023). Eplontersen is similar to inotersen, which was approved by the FDA in 2018 to treat hereditary transthyretin amyloidosis (Benson et al. 2018). Eplontersen has been approved for polyneuropathy of hereditary transthyretin-mediated amyloidosis in adults and received orphan drug designation. The most common AE were reduced vitamin A levels and vomiting.

## Cardiovascular and endocrine disorders

*Bexagliflozin* is the fifth sodium-glucose transporter 2 (SGLT-2) inhibitor, administered orally to diabetic patients to enhance glycemic control. Bexagliflozin has undergone investigation both as monotherapy (Halvorsen et al. 2019a, 2020) and in combination with metformin (Halvorsen et al. 2019b, 2023a, 2023b), demonstrating its effectiveness in improving glycemic control by lowering HbA1C levels (Halvorsen et al. 2019a, 2020, 2023a, 2023b). The most commonly observed AE associated with bexagliflozin treatment include female genital mycotic infections, urinary tract infections, and increased urination and are typical for this drug class. It is important to note that bexagliflozin is contraindicated in patients undergoing hemodialysis and is not recommended for individuals with type 1 diabetes or those with a low glomerular filtration rate (Hoy 2023a).

*Sotagliflozin* is a dual SGLT1 (intestinal) and SGLT2 (renal) inhibitor of glucose absorption and reabsorption, respectively. Clinical studies have revealed that initiating sotagliflozin therapy before discharge following hospitalization for worsening heart failure reduces cardiovascular death and heart failure (HF) events in patients with type 2 diabetes and chronic kidney disease (Bhatt et al. 2021; Pitt et al. 2023). Despite its positive impact on glycemic control, the FDA has not approved sotagliflozin for diabetes treatment due to concerns related to diabetic ketoacidosis (Markham and Keam 2019). The drug is approved to reduce the risk of cardiovascular death and worsening heart failure events in (1) patients with type 2 diabetes and chronic kidney disease with additional risk factors; and (2) patients with HF, with or without diabetes, regardless of ejection fraction. The latter approval is inexplicable, as no patients without diabetes were enrolled in the clinical studies (Packer 2023). While generally well-tolerated, sotagliflozin has AE similar to those observed with SGLT2 inhibitors (diarrhea, genital mycotic infections, volume depletion, and diabetic ketoacidosis) (Bhatt et al. 2021).

On the other hand, in 2023, FDA approved a groundbreaking therapy for the treatment of type 1 diabetes. *Donislecel-jujn* is the first allogeneic pancreatic islet cellular therapy obtained from deceased donors’ pancreatic cells, designed to address the challenges faced by diabetic patients unable to control glycated hemoglobin due to repeated episodes of hypoglycemia. Infused allogeneic islet cells secrete insulin and other pancreatic hormones, mimicking endogenous glucose control and homeostasis. Administrated through infusions into the hepatic portal vein, the treatment conveys insulin independence after 1–3 infusions (Alam et al. 2023). Moreover, 60% of islet-transplanted diabetic patients maintained insulin independence 5 years post-transplant (Qi et al. 2014). For optimal efficacy, donislecel should be used in conjunction with immunosuppressive therapy initiated before the procedure and continued on a long-term basis. The most frequently reported AE include nausea, fatigue, anemia, diarrhea, and abdominal pain.

A novel, first-in-class medication, *fezolinetant*, is designed to treat the vasomotor symptoms associated with menopause. This non-hormonal drug acts as a neurokinin 3 (NK3) receptor antagonist. Studies have shown that fezolinetant reduces both the frequency and severity of vasomotor symptoms compared to placebo (Johnson et al. 2023; Lederman et al. 2023). The daily oral administration of fezolinetant appears to be well tolerated with infrequent serious AE (Johnson et al. 2023). Abdominal pain, diarrhea, insomnia, back pain, hot flush, and hepatic transaminase elevation are reported as the most common AE of the therapy. Fezolinetant has received priority review designation.

Growth hormone deficiency is a rare condition characterized by insufficient growth hormone secretion, leading to short stature and delayed puberty. A novel recombinant human growth hormone, *somatrogon-ghla*, has been approved to treat growth failure in pediatric patients with growth hormone deficiency. This long-acting agent is injected subcutaneously once a week, thereby reducing injection frequency compared to currently available drugs. Clinical trials comparing the efficacy of once-weekly somatrogon with once-daily analogue of the somatotropin hormone somatropin have demonstrated similar effects on height velocity along with comparable safety and tolerability profiles for both treatments (Deal et al. 2022; Zadik et al. 2023). Reported AE include injection site reactions, nasopharyngitis, pyrexia, anemia, hypothyroidism, and oropharyngeal pain.

## Renal and gastrointestinal disorders

*Nedosiran* is a small interfering RNA designed to achieve hepatic lactate dehydrogenase (LDH) inhibition through suppression of the *LDHA* gene. As this enzyme plays a crucial role in the final step of oxalate synthesis, nedosiran lowers oxalate production thereby preventing the formation of kidney stones (Liu et al. 2022). This therapy has been approved for treating primary hyperoxaluria type 1 (PH1) in adults and high oxalate levels in children aged 9 years and older. Studies have shown that nedosiran injections effectively lower 24-h urinary oxalate compared to placebo (Baum et al. 2023; Goldfarb et al. 2023). The therapy has proven to be well-tolerated and safe, with injection site reactions reported as the only common AE effects. Nedosiran received breakthrough therapy designation.

An orally taken, dual antagonist targeting endothelin and angiotensin II receptor, *sparsentan*, has been approved for lowering proteinuria in adult patients with primary immunoglobulin A nephropathy at risk of rapid disease progression. Sparsentan caused a substantial reduction in proteinuria and maintained kidney function over 110 weeks compared to those receiving irbesartan (Heerspink et al. 2023; Rovin et al. 2023). Noteworthy, AE associated with sparsentan treatment include peripheral edema, hypotension, dizziness, hyperkalemia, and anemia. Due to potential severe AE, such as hepatotoxicity and embryo-fetal toxicity, sparsentan is exclusively available through Risk Evaluation and Mitigation Strategies. Sparsentan received accelerated approval.

Two novel drugs have been approved to treat ulcerative colitis in 2023. *Etrasimod* is a sphingosine 1-phosphate receptor modulator that targets receptors that play dominant roles in immune response, and pathologically particularly in immune-mediated diseases including ulcerative colitis, where T cells contribute to mucosal inflammation in the gastrointestinal tract. Clinical trials have demonstrated the efficacy of etrasimod in both inducing and maintaining treatment for moderate to severe ulcerative colitis (Sandborn et al. 2023). Etrasimod therapy has shown good tolerability, with common AE including headache, nausea, urinary tract infections, back pain, and dizziness (Silverberg et al. 2023). Of note, other sphingosine 1-phosphate receptor modulators have been approved earlier as treatments for multiple sclerosis (Kayki-Mutlu et al. 2022).

*Mirikizumab-mrkz*, an anti-interleukin (IL)-23 monoclonal antibody, has also been approved for the treatment of moderate-to-severe ulcerative colitis. Patients receiving mirikizumab demonstrated clinical remission and improved bowel-movement urgency in both induction and maintenance trials (D'Haens et al. 2023). The achievement of clinical response and remission was associated with an enhanced quality of life at week 12 and sustained through week 52 (Dubinsky et al. 2023). Opportunistic infections and cancer were reported in a small number of patients receiving mirikizumab (D'Haens et al. 2023). The most frequently observed AE are upper respiratory tract infections and arthralgia. Etrasimod is administered orally, while mirikizumab is given through intravenous infusion or subcutaneous injection.

## Infectious diseases

A sulbactam and durlobactam combination has received approval for the treatment of hospital-acquired bacterial pneumonia and ventilator-associated bacterial pneumonia (HABP/VABP) caused by *Acinetobacter*. Sulbactam has been used as a β-lactamase inhibitor, but it also has intrinsic antibacterial activity against *Acinetobacter* that is mediated by the inhibition of penicillin-binding proteins 1 and 3. The utility of sulbactam for *Acinetobacter* infections has been limited by its susceptibility to cleavage by numerous β-lactamases present in this species. Durlobactam inhibits serine β-lactamase, thus restoring sulbactam activity against *Acinetobacter* (El-Ghali et al. 2023). Sulbactam and durlobactam were non-inferior to colistin, each given as an add-on to imipenem with cilastatin. Sulbactam and durlobactam have demonstrated good tolerability and a lower risk of nephrotoxicity compared to colistin (Kaye et al. 2023; Watkins et al. 2023). This therapy has been granted fast track, qualified infectious disease product, and priority review designations.

In 2023, a novel oral antiviral therapy, comprising *nirmatrelvir* and ritonavir, has been approved for the treatment of COVID-19, marking the fourth drug approved by FDA for this indication. *Nirmatrelvir*, a 3C-like protease (3CL^PRO^) inhibitor, plays a crucial role in cleaving nonstructural proteins of the virus and inhibiting its replication. The oral availability of nirmatrelvir is advantageous, allowing for prescription to patients before the need for hospitalization. Ritonavir, an obsolete HIV protease inhibitor, increases the exposure of nirmatrelvir via inhibition of its inactivation by CYP3A4. Notably, older patients (65 years and above) receiving nirmatrelvir demonstrated lower rates of hospitalization and death due to COVID-19 compared to untreated ones (Arbel et al. 2022). The nirmatrelvir plus ritonavir combination lowers the risk of disease progression by 89% with no safety concerns (Hammond et al. 2022). The most frequently reported AE include dysgeusia and diarrhea. However, it is crucial to note that due to the many potential drug interactions mediated by CYP3A4, this therapy should be prescribed after a thorough review of all medications taken by the patient. Recognizing its present importance, this therapy has been granted emergency use authorization by the FDA for the treatment of mild-to-moderate COVID-19.

*Nirsevimab-alip*, a recombinant human IgG1ĸ monoclonal antibody, has received approval for the prevention of respiratory syncytial virus (RSV) lower respiratory tract disease in newborns and infants. It binds to the pre-fusion conformation of the RSV F protein. Administered intramuscularly as a single-dose for the entire RSV season, this long-acting agent protects infants from hospitalization throughout the 150 days of the RSV season (Griffin et al. 2020; Hammitt et al. 2022). Common AE include rash and injection site reactions. Nirsevimab received a fast-track designation.

*Rezafungin* has received approval for the treatment of candidemia and invasive candidiasis in patients aged 18 and above who have limited alternative treatment options. This echinocandin antifungal boasts extended pharmacokinetics and remarkable stability, making it well-suited for infrequent dosing intervals. With a half-life exceeding 130 h, it allows for weekly administration, presenting a convenient alternative to daily dosing. It is important to note that rezafungin is administered exclusively intravenously and does not attain therapeutic concentrations in the central nervous system, eyes, and urine. Clinical studies have convincingly demonstrated the efficacy of rezafungin, showing non-inferiority to caspofungin for primary endpoints such as day-14 global cure and 30-day all-cause mortality. Additionally, reports on the safety of rezafungin further support its use (Thompson et al. 2021, 2023). Common AE include hypokalemia, pyrexia, diarrhea, anemia, vomiting, nausea, hypomagnesemia, abdominal pain, constipation, and hypophosphatemia. The FDA granted rezafungin orphan drug, qualified infectious disease product, and fast track designations.

## Genetic disorders

In 2023, FDA approved nine drugs for genetic disorders, primarily rare diseases. *Velmanase alfa-tycv* which is a recombinant human lysosomal alpha-mannosidase is indicated to treat alpha-mannosidosis. It is the first enzyme-replacement therapy to target the non-neurological symptoms associated with the disease. This rare autosomal recessive lysosomal storage disorder arises from genetic mutations in the *MAN2B1* gene, resulting in the inadequate production of the alpha-mannosidase enzyme. This enzyme plays a crucial role in breaking down complex sugars. Consequently, its deficiency leads to the accumulation of mannose-rich oligosaccharides, contributing to progressive neuromuscular and skeletal deterioration (Guffon et al. 2023). Velmanase treatment has been demonstrated to be effective in reducing serum oligosaccharide levels. Notably, best benefits were observed when therapy was initiated during childhood (Borgwardt et al. 2018). The treatment involves weekly injections. Common AE include hypersensitivity reactions, such as anaphylaxis, as well as nasopharyngitis, pyrexia, headache, and arthralgia. The FDA granted velmanase orphan drug designation.

A potent and selective inhibitor of phosphoinositide 3-kinase δ (PI3Kδ), *leniolisib*, was approved for the treatment of activated phosphoinositide 3-kinase delta syndrome. Remarkably, it stands as the first therapy to address this disorder in both adults and pediatric patients aged 12 and above. The syndrome is caused by mutations in PIK3CD or PIK3R1 genes, which together encode the heterodimer PI3Kδ. Consequently, augmented PI3Kδ activity ensues, leading to immune dysfunction and increased susceptibility to infections (Duggan and Al-Salama 2023). Clinical trials have shown that leniolisib, administered twice daily for 12 weeks, enhances parameters such as the lymph node size and the percentage of naïve B cells in peripheral blood over placebo (Rao et al. 2023). AE include headache, sinusitis, and atopic dermatitis. Leniolisib received orphan drug designation, rare pediatric disease designation, and priority review.

Fabry disease is a rare genetic disorder linked to alpha-galactosidase A deficiency. *Pegunigalsidase α-iwxj* is a recombinant form of human α-galactosidase-A indicated as long-term enzyme replacement therapy in the treatment of Fabry disease. Notably, its plant cell-based protein expression system and pegylated structure contribute to enhanced stability and half-life, decreased immunogenicity, and increased efficacy compared to similar available agents (Schiffmann et al. 2019). Clinical trials, focusing on renal and cardiac function, adverse events, and the presence of IgG antidrug antibodies, have consistently reported the safety and effectiveness of pegunigalsidase α when administered through intravenous infusion every other week (Schiffmann et al. 2019; Linhart et al. 2023). The most frequently observed AE include infusion-associated reactions, nasopharyngitis, pain in extremity, and sinusitis. Pegunigalsidase α has received fast track designation and priority review.

Fibrodysplasia ossificans progressiva (FOP), an exceptionally rare genetic disorder, is attributable to mutations in the ACVR1/ALK2 gene, leading to progressive heterotopic ossification. This process entails the transformation of connective tissues into bone tissue. *Palovarotene*, a selective agonist of the retinoic acid receptor gamma (RARγ), received approval for mitigating heterotopic ossification in patients afflicted by FOP. It is the first medication approved for treating FOP with eligibility starting at 8 years for females and 10 years for males. Clinical studies assessing palovarotene have demonstrated its efficacy in reducing heterotopic ossification by impacting lower vertebral bone mineral density and content, and strength while increasing vertebral fracture resistance (Pignolo et al. 2023). The most commonly reported AE of palovarotene include dry skin, lip dryness, arthralgia, pruritus, rash, alopecia, erythema, skin exfoliation, musculoskeletal pain, myalgia, dry eye, hypersensitivity, peripheral edema, and fatigue. FDA granted palovarotene priority review and orphan drug designation.

*Pozelimab-bbfg* has been approved for the treatment of another ultra-rare hereditary disease known as CD55-deficient protein-losing enteropathy also known as CHAPLE disease. CHAPLE, which stands for CD55 deficiency with hyper-activation of complement, angiopathic thrombosis, and severe protein-losing enteropathy, results from CD55 deficiency leading to a hyperactivated complement system that mediates the formation of the membrane-attacking complex (Ozen et al. 2017). Pozelimab is a human monoclonal IgG4 antibody targeting the terminal complement protein C5. It marks the first treatment indicated for CHAPLE disease and is available for patients starting from 1 year of age. The initial dose is administered intravenously, followed by weekly subcutaneous injections. Clinical evidence has demonstrated that pozelimab administration effectively inhibits intravascular hemolysis and is generally well-tolerated (Jang et al. 2021). Common AE to the therapy include upper respiratory tract infections, fractures, urticaria, and alopecia. It is crucial to note that pozelimab carries a boxed warning for life-threatening and fatal meningococcal infections. The FDA has granted pozelimab fast track, orphan drug, priority review and rare pediatric disease designations.

*Cipaglucosidase α-atga* has received approval for treating late-onset Pompe disease in adults weighing at least 40 kg, particularly those who do not show improvement on their existing enzyme replacement therapy. This rare genetic metabolic disorder is linked to a deficiency of human acid α-glucosidase. Cipaglucosidase α is a recombinant human α-glucosidase, modified to enable intracellular uptake via mannose 6 receptors. The drug is administered every other week intravenously together with miglustat, a small molecule that stabilizes the conformation of the enzyme. Cipaglucosidase α plus miglustat treatment has been shown to improve the 6-min walking distance and biomarkers linked to Pompe disease and has a safe and well tolerated profile (Byrne et al. 2023). Although this combination was not found to be superior over alglucosidase α plus placebo group in week 52 (Schoser et al. 2021), longer treatment with this combination for up to 104 weeks has been reported to maintain a durable effect (Blair 2023a). Common AE include headache, diarrhea, fatigue, nausea, abdominal pain, and pyrexia. Hypersensitivity reactions, including anaphylaxis, are also potential risks associated with this treatment. It received orphan drug and breakthrough designations.

Two gene therapies have been approved in 2023 for the treatment of sickle cell disease in patients aged 12 years or older with a history of vaso-occlusive events. Sickle cell disease is an autosomal recessive condition characterized by a mutation in the β-globin gene, leading to the formation of hemoglobin S. This abnormal hemoglobin variant causes the red cells to become rigid and take on a sickle shape. *Lovotibeglogene autotemcel*, an autologous CD34^+^ cell therapy, involves hematopoietic stem and progenitor cells encoding a functional copy of the β-globin gene. This innovative therapy aims to address the underlying genetic cause of sickle cell disease by providing patients with a corrected and functional version of the affected gene. A single intravenous administration of lovotibeglogene has demonstrated the ability to sustain the formation of anti-sickling hemoglobin, known as HbAT87Q. This modified adult hemoglobin involves an amino acid substitution, specifically threonine to glutamine at position 87. The therapy has been shown to mitigate impaired hemolysis and reduce vaso-occlusive events (Kanter et al. 2022). The most frequently observed AE include stomatitis, thrombocytopenia, neutropenia, febrile neutropenia, anemia, and leukopenia. The FDA has granted lovotibeglogene orphan drug designation, fast track designation, and rare pediatric disease designation.

The other sickle cell therapy is *exagamglogene autotemcel*, the first treatment utilizing CRISPR/Cas9 genome editing. Clinical trials have demonstrated a reduction in vaso-occlusive crises in patients treated with exagamglogene autotemcel. The therapy has also been reported to be associated with an independence from transfusion in the patients with β-thalassemia (Kingwell 2023), for which it is approved in the UK. Following myeloablative conditioning, the drug is administered intravenously as a suspension of hematopoietic stem and progenitor cells capable of producing fetal hemoglobin. To this end, cells are edited by CRISPR/Cas9 at the erythroid specific enhancer region of the *BCL11A* gene. Neutropenia, thrombocytopenia, leukopenia, anemia, lymphopenia, mucositis, and decreased appetite are among the most common AE. It has received orphan drug, fast track, and rare pediatric disease designations.

## Dermatology

*Ritlecitinib* has received approval for the treatment of the immune disorder alopecia areata in adults and adolescents aged 12 years and older. It is a highly selective and irreversible dual inhibitor targeting Janus kinase 3 (JAK3) and TEC kinases and has immunosuppressive effects. Notably, it is the first oral drug in its class. Studies have shown that ritlecitinib effectively reduces the severity of alopecia and is well tolerated (Hordinsky et al. 2023; King et al. 2023). Most common AE are headache, diarrhea, acne, rash, urticaria, pyrexia, atopic dermatitis, blood creatine phosphokinase increased, herpes zoster, red blood cell count decreased, and stomatitis.

A gene therapy, *beremagene geperpavec-svdt*, has been approved to treat skin wounds in patients with dystrophic epidermolysis bullosa. This ultra-rare and life-threatening genetic disorder stems from mutations in the collagen type VII alpha 1 chain (*COL7A1*) gene, which encodes collagen VII (COL7). COL7 plays a crucial role as a component of anchoring fibrils essential for dermal-epidermal cohesion. Consequently, mutations in this gene lead to the loss of structural integrity in anchoring fibrils in the dermis (Dhillon 2023a). Beremagene geperpavec comprises replication-defective herpes simplex virus type 1 (HSV-1) carrying a *COL7A1* gene, incorporated into an excipient gel. This gel is topically applied to the wounds once a week to restore COL7 levels. The repeated administration of beremagene geperpavec resulted in complete wound healing at 3 and 6 months (Guide et al. 2022). The therapy has also demonstrated efficacy in reducing wound surface area, and in improving the duration of and time to wound closure (Gurevich et al. 2022). AE include pruritus, chills, and mild systemic effects. Beremagene geperpavec has received orphan drug designation.

A second drug approved for the treatment of both dystrophic and junctional epidermolysis bullosa is *birch triterpenes*. This therapy is intended for use in patients aged 6 months and older and is administered topically. The topical gel comprises extracts from birch bark containing natural triterpenes, such as betulin, betulinic acid, erythrodiol, lupeol, and oleanolic acid. Research has revealed that the application of topical birch triterpenes enhances wound healing within 45 days. Importantly, the incidence of AE, including infections, was found to be similar to that observed with control gel treatment (Kern et al. 2023). The FDA granted birch triterpenes orphan drug, fast track, and priority review designations. The approval of a plant extract as a medicine and not as a dietary supplement is a rare occurrence for the FDA, but it is understandable considering the severity of the medical indication.

A monoclonal antibody *bimekizumab-bkzx* was approved to treat plaque psoriasis. This dual inhibitor selectively targets interleukin 17A (IL-17A) and interleukin 17F (IL-17F), which play crucial roles in inflammatory processes (Reis et al. 2019). The efficacy of bimekizumab has been substantiated through the American College of Rheumatology response criteria and the Psoriasis Area and Severity Index, demonstrating consistent maintenance from week 16 to week 52 of treatment (Ritchlin et al. 2023). In another clinical trial, complete skin clearance was achieved at week 48 and was sustained at week 96 with bimekizumab therapy favorably comparing to secukinumab. AE include nasopharyngitis, oral candidiasis, and urinary tract infection (Strober et al. 2023).

## Ophthalmology

Three drugs for ophthalmic diseases have received approval in 2023. *Perfluorohexyloctane* was approved to address signs and symptoms of dry eye disease. It is a semifluorinated alkane—a chemically inert, slightly amphiphilic compound. Being a completely non-aqueous liquid, it eliminates the possibility of microbial growth, obviating the need for added preservatives. Studies have demonstrated that perfluorohexyloctane therapy reduces the signs and symptoms of dry eye disease compared to a hypotonic saline control. Moreover, it is well-tolerated and exhibits an AE profile similar to that of saline (Sheppard et al. 2023; Tauber et al. 2023). The most common ocular AE reported was blurred vision.

*Lotilaner*, an antiparasitic agent, is indicated for the treatment of Demodex blepharitis caused by Demodex mites. Originally employed for veterinary purposes to combat flea and tick infestations, lotilaner operates as a selective inhibitor of the γ-aminobutyric acid-gated chloride (GABA-Cl) channel specific to insects. The S-enantiomer is the active component in the drug product which is the first-in-class medication for this ocular disease. Twice-daily treatment with lotilaner ophthalmic solution has demonstrated significant reduction in collarettes in the upper eyelid and effective mite eradication (Gaddie et al. 2023; Yeu et al. 2023). This medication is recognized for its safety and high tolerance levels, with the most frequent adverse effects reported being site stinging and burning.

*Avacincaptad pegol*, a complement C5 inhibitor, is indicated for the treatment of an advanced form of age-related macular degeneration called geographic atrophy, which leads to bilateral and irreversible vision loss over time. Intravitreal injection of avacincaptad was reported to reduce geographic atrophy in eyes over a 12-month period (Jaffe et al. 2021). The therapy was also demonstrated to mediate macular neovascularization more frequently compared to sham control and to be well tolerated over 18 months with ocular side effects (Patel et al. 2023). Most common AE include conjunctival hemorrhage, increased intraocular pressure, blurred vision, and neovascular age-related macular degeneration. The FDA granted avacincaptad pegol fast track, breakthrough therapy designations and priority review.

## Diagnostic agents

A radioactive diagnostic agent, flotufolastat ^18^F, has been approved to be used in positron emission tomography imaging to visualize prostate-specific membrane antigen-positive lesions in patients with prostate cancer with suspected recurrence or metastasis. Flotufolastat was reported to have high specificity and an excellent safety profile (Surasi et al. 2023). It is administered as an intravenous bolus injection. AE include diarrhea, blood pressure increase, and injection site pain.

## General trends and conclusions

The number of novel drugs approved by the FDA in 2023 was similar to that in the past several years (Kayki-Mutlu and Michel 2021; Kayki-Mutlu et al. 2022), except the pandemic-related dent observed in 2022 (Kayki-Mutlu et al. 2023). The share of small molecules among new drug approvals has continuously declined in the past 4 years from 70% in 2020 to 49% in 2023. Concomitantly, the share of antibodies (22–27%) and of peptides and proteins other than antibodies (15–19%) remained stable. Of note, the share of nucleic acid, gene, and cell therapy products increased from 4% in 2020 and 2021 to now 20%. Regarding degree of innovation, we did not observe a consistent trend with first-in-indication ranging from 2 to 11% and first-in-class from 28 to 54% over time; the share of next-in-class medication also did not exhibit a clear trend ranging from 46 to 60% in the past 4 years.

By treatment area, rare diseases/orphan drugs (54%) and cancer drugs (23%) were once again the most prevalent (and partly overlapping) therapeutic areas with neither exhibiting a clear trend over the past 4 years. By coincidence, three approvals target major medical problems predominantly or exclusively found in women. Fezolinetant has been approved for menopause-related vasomotor symptoms, zuranolone for postpartum depression, and elecestrant for advanced or metastatic breast cancer in postmenopausal women (and less frequently in adult men).

2023 marks the beginning of the availability of immunization options against the RSV-related lower respiratory tract disease in newborns and infants. Besides the passive immunization using nirsevimab, they include two active vaccines, not covered in this review. The approval of the sickle cell therapeutic exagamglogene autotemcel marks the beginning of an entire new treatment era—that of genome editing.

The various special processing procedures continued to be used at high rates: In 2023, 41% of approved drugs were granted fast track status as compared to 32% in 2020–2022; a breakthrough therapy designation was provided in 42% in 2020 and has continuously declined to 22% in 2023. In contrast, priority review (52–57%) and accelerated approval (16–23%) remained stable over the past 4 years. The availability of these accelerated regulatory pathways may also have provided incentives to focus clinical development projects on conditions where such designations are likely.

## Data Availability

No datasets were generated or analysed during the current study.

## References

[CR1] Abeti R, Baccaro A, Esteras N, Giunti P (2018). Novel Nrf2-inducer prevents mitochondrial defects and oxidative stress in Friedreich’s ataxia models. Front Cell Neurosci.

[CR2] Adams D, Ando Y, Beirao JM, Coelho T, Gertz MA, Gillmore JD, Hawkins PN, Lousada I, Suhr OB, Merlini G (2021). Expert consensus recommendations to improve diagnosis of ATTR amyloidosis with polyneuropathy. J Neurol.

[CR3] Alam S, Khan SJ, Lee CYF, Zaidi SAT, Murtaza SF (2023). Type 1 diabetes mellitus management and islet cell therapy: a new chapter in patient care. Cureus.

[CR4] Ambinder AJ, Levis M (2021). Potential targeting of FLT3 acute myeloid leukemia. Haematologica.

[CR5] Andrikopoulou A, Chatzinikolaou S, Panourgias E, Kaparelou M, Liontos M, Dimopoulos MA, Zagouri F (2022). The emerging role of capivasertib in breast cancer. Breast.

[CR6] Arbel R, Wolff Sagy Y, Hoshen M, Battat E, Lavie G, Sergienko R, Friger M, Waxman JG, Dagan N, Balicer R, Ben-Shlomo Y, Peretz A, Yaron S, Serby D, Hammerman A, Netzer D (2022). Nirmatrelvir use and severe Covid-19 outcomes during the Omicron Surge. N Engl J Med.

[CR7] Bardia A, Kaklamani V, Wilks S, Weise A, Richards D, Harb W, Osborne C, Wesolowski R, Karuturi M, Conkling P, Bagley RG, Wang Y, Conlan MG, Kabos P (2021). Phase I study of elacestrant (RAD1901), a novel selective estrogen receptor degrader, in ER-positive, HER2-negative advanced breast cancer. J Clin Oncol.

[CR8] Baum MA, Langman C, Cochat P, Lieske JC, Moochhala SH, Hamamoto S, Satoh H, Mourani C, Ariceta G, Torres A, Wolley M, Belostotsky V, Forbes TA, Groothoff J, Hayes W, Tönshoff B, Takayama T, Rosskamp R, Russell K, Zhou J, Amrite A, Hoppe B (2023). PHYOX2: a pivotal randomized study of nedosiran in primary hyperoxaluria type 1 or 2. Kidney Int.

[CR9] Benson MD, Waddington-Cruz M, Berk JL, Polydefkis M, Dyck PJ, Wang AK, Plante-Bordeneuve V, Barroso FA, Merlini G, Obici L, Scheinberg M, Brannagan TH, Litchy WJ, Whelan C, Drachman BM, Adams D, Heitner SB, Conceicao I, Schmidt HH, Vita G, Campistol JM, Gamez J, Gorevic PD, Gane E, Shah AM, Solomon SD, Monia BP, Hughes SG, Kwoh TJ, McEvoy BW, Jung SW, Baker BF, Ackermann EJ, Gertz MA, Coelho T (2018). Inotersen treatment for patients with hereditary transthyretin amyloidosis. N Engl J Med.

[CR10] Beumer JH, Foldi J (2023). Pharmacology and pharmacokinetics of elacestrant. Cancer Chemother Pharmacol.

[CR11] Bhatt DL, Szarek M, Pitt B, Cannon CP, Leiter LA, McGuire DK, Lewis JB, Riddle MC, Inzucchi SE, Kosiborod MN, Cherney DZI, Dwyer JP, Scirica BM, Bailey CJ, Díaz R, Ray KK, Udell JA, Lopes RD, Lapuerta P, Steg PG (2021). Sotagliflozin in patients with diabetes and chronic kidney disease. N Engl J Med.

[CR12] Bidard FC, Kaklamani VG, Neven P, Streich G, Montero AJ, Forget F, Mouret-Reynier MA, Sohn JH, Taylor D, Harnden KK, Khong H, Kocsis J, Dalenc F, Dillon PM, Babu S, Waters S, Deleu I, Garcia Saenz JA, Bria E, Cazzaniga M, Lu J, Aftimos P, Cortes J, Liu S, Tonini G, Laurent D, Habboubi N, Conlan MG, Bardia A (2022). Elacestrant (oral selective estrogen receptor degrader) versus standard endocrine therapy for estrogen receptor-positive, human epidermal growth factor receptor 2-negative advanced breast cancer: results from the randomized phase III EMERALD trial. J Clin Oncol.

[CR13] Blair HA (2022). Valoctocogene roxaparvovec: first approval. Drugs.

[CR14] Blair HA (2023). Cipaglucosidase alfa: first approval. Drugs.

[CR15] Blair HA (2023). Efbemalenograstim alfa: first approval. Drugs.

[CR16] Blair HA (2023). Tofersen: first approval. Drugs.

[CR17] Blayney DW, Schwartzberg L (2022). Chemotherapy-induced neutropenia and emerging agents for prevention and treatment: a review. Cancer Treat Rev.

[CR18] Borgwardt L, Guffon N, Amraoui Y, Dali CI, De Meirleir L, Gil-Campos M, Heron B, Geraci S, Ardigò D, Cattaneo F, Fogh J, Van den Hout JMH, Beck M, Jones SA, Tylki-Szymanska A, Haugsted U, Lund AM (2018). Efficacy and safety of Velmanase alfa in the treatment of patients with alpha-mannosidosis: results from the core and extension phase analysis of a phase III multicentre, double-blind, randomised, placebo-controlled trial. J Inherit Metab Dis.

[CR19] Brodsky RA (2014). Paroxysmal nocturnal hemoglobinuria. Blood.

[CR20] Byrne BJ, Schoser B, Kishnani PS, Bratkovic D, Clemens PR, Goker-Alpan O, Ming X, Roberts M, Vorgerd M, Sivakumar K, van der Ploeg AT, Goldman M, Wright J, Holdbrook F, Jain V, Benjamin ER, Johnson F, Das SS, Wasfi Y, Mozaffar T (2023). Long-term safety and efficacy of cipaglucosidase alfa plus miglustat in individuals living with Pompe disease: an open-label phase I/II study (ATB200-02). J Neurol.

[CR21] Chifotides HT, Bose P, Verstovsek S (2022). Momelotinib: an emerging treatment for myelofibrosis patients with anemia. J Hematol Oncol.

[CR22] Coelho T, Waddington Cruz M, Chao CC, Parman Y, Wixner J, Weiler M, Barroso FA, Dasgupta NR, Jung SW, Schneider E, Viney NJ, Dyck PJB, Ando Y, Gillmore JD, Khella S, Gertz MA, Obici L, Berk JL (2023). Characteristics of patients with hereditary transthyretin amyloidosis-polyneuropathy (ATTRv-PN) in NEURO-TTRansform, an open-label phase 3 study of eplontersen. Neurol Ther.

[CR23] Couzin-Frankel J, Piller C (2022). Alzheimer’s drug stirs excitement-and concerns. Science.

[CR24] Crawford J, Oswalt C (2023). The impact of new and emerging agents on outcomes for febrile neutropenia: addressing clinical gaps. Curr Opin Oncol.

[CR25] Crees ZD, Rettig MP, Bashey A, Devine SM, Jaglowski S, Wan F, Zhou A, Harding M, Vainstein-Haras A, Sorani E, Gliko-Kabir I, Grossman BJ, Westervelt P, DiPersio JF, Uy GL (2023). Hematopoietic stem cell mobilization for allogeneic stem cell transplantation by motixafortide, a novel CXCR4 inhibitor. Blood Adv.

[CR26] Crees ZD, Rettig MP, Jayasinghe RG, Stockerl-Goldstein K, Larson SM, Arpad I, Milone GA, Martino M, Stiff P, Sborov D, Pereira D, Micallef I, Moreno-Jimenez G, Mikala G, Coronel MLP, Holtick U, Hiemenz J, Qazilbash MH, Hardy N, Latif T, Garcia-Cadenas I, Vainstein-Haras A, Sorani E, Gliko-Kabir I, Goldstein I, Ickowicz D, Shemesh-Darvish L, Kadosh S, Gao F, Schroeder MA, Vij R, DiPersio JF (2023). Motixafortide and G-CSF to mobilize hematopoietic stem cells for autologous transplantation in multiple myeloma: a randomized phase 3 trial. Nat Med.

[CR27] Dayalan Naidu S, Dinkova-Kostova AT (2023). Omaveloxolone (Skyclarys(TM)) for patients with Friedreich’s ataxia. Trends Pharmacol Sci.

[CR28] Deal CL, Steelman J, Vlachopapadopoulou E, Stawerska R, Silverman LA, Phillip M, Kim HS, Ko C, Malievskiy O, Cara JF, Roland CL, Taylor CT, Valluri SR, Wajnrajch MP, Pastrak A, Miller BS (2022). Efficacy and safety of weekly somatrogon vs daily somatropin in children with growth hormone deficiency: a phase 3 study. J Clin Endocrinol Metab.

[CR29] D'Haens G, Dubinsky M, Kobayashi T, Irving PM, Howaldt S, Pokrotnieks J, Krueger K, Laskowski J, Li X, Lissoos T, Milata J, Morris N, Arora V, Milch C, Sandborn W, Sands BE (2023). Mirikizumab as induction and maintenance therapy for ulcerative colitis. N Engl J Med.

[CR30] Dhillon S (2020). Daprodustat: first approval. Drugs.

[CR31] Dhillon S (2023). Beremagene geperpavec: first approval. Drugs.

[CR32] Dhillon S (2023). Elranatamab: first approval. Drugs.

[CR33] Dhillon S (2023). Zavegepant: first approval. Drugs.

[CR34] Dubinsky MC, Jairath V, Feagan BG, Naegeli AN, Tuttle J, Morris N, Shan M, Arora V, Lissoos T, Agada N, Hibi T, Sands BE (2023) Changes in health-related quality of life and associations with improvements in clinical efficacy: a phase 2 study of mirikizumab in patients with ulcerative colitis. BMJ Open Gastroenterol 10. 10.1136/bmjgast-2023-001115 10.1136/bmjgast-2023-001115 PMC10069555 37001911

[CR35] Duggan S, Al-Salama ZT (2023). Leniolisib: first approval. Drugs.

[CR36] Dyrbekk APH, Warsame AA, Suhrke P, Ludahl MO, Moe JO, Eide IJZ, Lund-Iversen M, Brustugun OT (2023). Evaluation of ROS1 expression and rearrangements in a large cohort of early-stage lung cancer. Diagn Pathol.

[CR37] El-Ghali A, Kunz Coyne AJ, Caniff K, Bleick C, Rybak MJ (2023). Sulbactam-durlobactam: a novel β-lactam-β-lactamase inhibitor combination targeting carbapenem-resistant Acinetobacter baumannii infections. Pharmacotherapy.

[CR38] Engelberts PJ, Hiemstra IH, de Jong B, Schuurhuis DH, Meesters J, Beltran Hernandez I, Oostindie SC, Neijssen J, van den Brink EN, Horbach GJ, Verploegen S, Labrijn AF, Salcedo T, Schuurman J, Parren P, Breij ECW (2020). DuoBody-CD3xCD20 induces potent T-cell-mediated killing of malignant B cells in preclinical models and provides opportunities for subcutaneous dosing. EBioMedicine.

[CR39] Fassel H, McGuinn C (2021). Haemophilia: factoring in new therapies. Br J Haematol.

[CR40] Frampton JE (2023). Epcoritamab: first approval. Drugs.

[CR41] Gaddie IB, Donnenfeld ED, Karpecki P, Vollmer P, Berdy GJ, Peterson JD, Simmons B, Edell ARP, Whitson WE, Ciolino JB, Baba SN, Holdbrook M, Trevejo J, Meyer J, Yeu E (2023). Lotilaner ophthalmic solution 0.25% for Demodex blepharitis: randomized, vehicle-controlled, multicenter, phase 3 trial (saturn-2). Ophthalmology.

[CR42] Gauci ML, Aristei C, Becker JC, Blom A, Bataille V, Dreno B, Del Marmol V, Forsea AM, Fargnoli MC, Grob JJ, Gomes F, Hauschild A, Hoeller C, Harwood C, Kelleners-Smeets N, Kaufmann R, Lallas A, Malvehy J, Moreno-Ramirez D, Peris K, Pellacani G, Saiag P, Stratigos AJ, Vieira R, Zalaudek I, van Akkooi ACJ, Lorigan P, Garbe C, Lebbe C, European Dermatology Forum tEAoD-O, the European Organization for R, Treatment of C (2022). Diagnosis and treatment of Merkel cell carcinoma: European consensus-based interdisciplinary guideline - update 2022. Eur J Cancer.

[CR43] Giralt S, Costa L, Schriber J, Dipersio J, Maziarz R, McCarty J, Shaughnessy P, Snyder E, Bensinger W, Copelan E, Hosing C, Negrin R, Petersen FB, Rondelli D, Soiffer R, Leather H, Pazzalia A, Devine S (2014). Optimizing autologous stem cell mobilization strategies to improve patient outcomes: consensus guidelines and recommendations. Biol Blood Marrow Transplant.

[CR44] Goldfarb DS, Lieske JC, Groothoff J, Schalk G, Russell K, Yu S, Vrhnjak B (2023). Nedosiran in primary hyperoxaluria subtype 3: results from a phase I, single-dose study (PHYOX4). Urolithiasis.

[CR45] Gounder M, Ratan R, Alcindor T, Schoffski P, van der Graaf WT, Wilky BA, Riedel RF, Lim A, Smith LM, Moody S, Attia S, Chawla S, D'Amato G, Federman N, Merriam P, Van Tine BA, Vincenzi B, Benson C, Bui NQ, Chugh R, Tinoco G, Charlson J, Dileo P, Hartner L, Lapeire L, Mazzeo F, Palmerini E, Reichardt P, Stacchiotti S, Bailey HH, Burgess MA, Cote GM, Davis LE, Deshpande H, Gelderblom H, Grignani G, Loggers E, Philip T, Pressey JG, Kummar S, Kasper B (2023). Nirogacestat, a gamma-secretase inhibitor for desmoid tumors. N Engl J Med.

[CR46] Griffin MP, Yuan Y, Takas T, Domachowske JB, Madhi SA, Manzoni P, Simões EAF, Esser MT, Khan AA, Dubovsky F, Villafana T, DeVincenzo JP (2020). Single-dose nirsevimab for prevention of RSV in preterm infants. N Engl J Med.

[CR47] Guffon N, Konstantopoulou V, Hennermann JB, Muschol N, Bruno I, Tummolo A, Ceravolo F, Zardi G, Ballabeni A, Lund A (2023). Long-term safety and efficacy of velmanase alfa treatment in children under 6 years of age with alpha-mannosidosis: a phase 2, open label, multicenter study. J Inherit Metab Dis.

[CR48] Guglieri M, Clemens PR, Perlman SJ, Smith EC, Horrocks I, Finkel RS, Mah JK, Deconinck N, Goemans N, Haberlova J, Straub V, Mengle-Gaw LJ, Schwartz BD, Harper AD, Shieh PB, De Waele L, Castro D, Yang ML, Ryan MM, McDonald CM, Tulinius M, Webster R, McMillan HJ, Kuntz NL, Rao VK, Baranello G, Spinty S, Childs AM, Sbrocchi AM, Selby KA, Monduy M, Nevo Y, Vilchez-Padilla JJ, Nascimento-Osorio A, Niks EH, de Groot IJM, Katsalouli M, James MK, van den Anker J, Damsker JM, Ahmet A, Ward LM, Jaros M, Shale P, Dang UJ, Hoffman EP (2022). Efficacy and Safety of vamorolone vs placebo and prednisone among boys with Duchenne muscular dystrophy: a randomized clinical trial. JAMA Neurol.

[CR49] Guide SV, Gonzalez ME, Bağcı IS, Agostini B, Chen H, Feeney G, Steimer M, Kapadia B, Sridhar K, Quesada Sanchez L, Gonzalez F, Van Ligten M, Parry TJ, Chitra S, Kammerman LA, Krishnan S, Marinkovich MP (2022). Trial of beremagene geperpavec (B-VEC) for dystrophic epidermolysis bullosa. N Engl J Med.

[CR50] Gunduz-Bruce H, Takahashi K, Huang MY (2022). Development of neuroactive steroids for the treatment of postpartum depression. J Neuroendocrinol.

[CR51] Gurevich I, Agarwal P, Zhang P, Dolorito JA, Oliver S, Liu H, Reitze N, Sarma N, Bagci IS, Sridhar K, Kakarla V, Yenamandra VK, O'Malley M, Prisco M, Tufa SF, Keene DR, South AP, Krishnan SM, Marinkovich MP (2022). In vivo topical gene therapy for recessive dystrophic epidermolysis bullosa: a phase 1 and 2 trial. Nat Med.

[CR52] Haider MU, Furqan M, Mehmood Q (2023). Daprodustat: a potential game-changer in renal anemia therapy-A perspective. Front Pharmacol.

[CR53] Halvorsen YC, Walford GA, Massaro J, Aftring RP, Freeman MW (2019). A 96-week, multinational, randomized, double-blind, parallel-group, clinical trial evaluating the safety and effectiveness of bexagliflozin as a monotherapy for adults with type 2 diabetes. Diabetes Obes Metab.

[CR54] Halvorsen YD, Lock JP, Zhou W, Zhu F, Freeman MW (2019). A 24-week, randomized, double-blind, active-controlled clinical trial comparing bexagliflozin with sitagliptin as an adjunct to metformin for the treatment of type 2 diabetes in adults. Diabetes Obes Metab.

[CR55] Halvorsen YD, Walford G, Thurber T, Russell H, Massaro M, Freeman MW (2020). A 12-week, randomized, double-blind, placebo-controlled, four-arm dose-finding phase 2 study evaluating bexagliflozin as monotherapy for adults with type 2 diabetes. Diabetes Obes Metab.

[CR56] Halvorsen YD, Conery AL, Lock JP, Zhou W, Freeman MW (2023). Bexagliflozin as an adjunct to metformin for the treatment of type 2 diabetes in adults: a 24-week, randomized, double-blind, placebo-controlled trial. Diabetes Obes Metab.

[CR57] Halvorsen YD, Lock JP, Frias JP, Tinahones FJ, Dahl D, Conery AL, Freeman MW (2023). A 96-week, double-blind, randomized controlled trial comparing bexagliflozin to glimepiride as an adjunct to metformin for the treatment of type 2 diabetes in adults. Diabetes Obes Metab.

[CR58] Hammitt LL, Dagan R, Yuan Y, Baca Cots M, Bosheva M, Madhi SA, Muller WJ, Zar HJ, Brooks D, Grenham A, Wählby Hamrén U, Mankad VS, Ren P, Takas T, Abram ME, Leach A, Griffin MP, Villafana T (2022). Nirsevimab for prevention of RSV in healthy late-preterm and term infants. N Engl J Med.

[CR59] Hammond J, Leister-Tebbe H, Gardner A, Abreu P, Bao W, Wisemandle W, Baniecki M, Hendrick VM, Damle B, Simón-Campos A, Pypstra R, Rusnak JM (2022). Oral nirmatrelvir for high-risk, nonhospitalized adults with Covid-19. N Engl J Med.

[CR60] Han B, Ji J, Zhang B, Bai H, Zhou D, Feng F, Huang Y, Zhu H, Chen L, Wu Z, Jiang X, Li X, Jia Q, Chang Q, Pan H, Peng H, Zheng W, Huang H, Chen Z, Yang C, Chen M, Du B, Zhang S (2022). The first successful expanded compassionate use of Iptacopan in a patient with paroxysmal nocturnal hemoglobinuria. Ann Hematol.

[CR61] Han J, Zeng N, Tian K, Liu Z, She L, Wang Z, He J, Chen N (2023). First-line immunotherapy combinations for recurrent or metastatic nasopharyngeal carcinoma: an updated network meta-analysis and cost-effectiveness analysis. Head Neck.

[CR62] Hatashima A, Karami M, Shadman M (2022). Approved and emerging Bruton’s tyrosine kinase inhibitors for the treatment of chronic lymphocytic leukemia. Expert Opin Pharmacother.

[CR63] Heerspink HJL, Radhakrishnan J, Alpers CE, Barratt J, Bieler S, Diva U, Inrig J, Komers R, Mercer A, Noronha IL, Rheault MN, Rote W, Rovin B, Trachtman H, Trimarchi H, Wong MG, Perkovic V (2023). Sparsentan in patients with IgA nephropathy: a prespecified interim analysis from a randomised, double-blind, active-controlled clinical trial. Lancet.

[CR64] Heo YA (2023). Omidubicel: first approval. Mol Diagn Ther.

[CR65] Heo YA (2023). Zuranolone: first approval. Drugs.

[CR66] Hordinsky M, Hebert AA, Gooderham M, Kwon O, Murashkin N, Fang H, Harada K, Law E, Wajsbrot D, Takiya L, Zwillich SH, Wolk R, Tran H (2023). Efficacy and safety of ritlecitinib in adolescents with alopecia areata: results from the ALLEGRO phase 2b/3 randomized, double-blind, placebo-controlled trial. Pediatr Dermatol.

[CR67] Hoy SM (2023). Bexagliflozin: first approval. Drugs.

[CR68] Hoy SM (2023). Elacestrant: first approval. Drugs.

[CR69] Hoy SM (2023). Lecanemab: first approval. Drugs.

[CR70] Hoy SM (2023). Motixafortide: first approval. Drugs.

[CR71] Hoy SM (2023). Rozanolixizumab: first approval. Drugs.

[CR72] Hudu SA, Elmigdadi F, Qtaitat AA, Almehmadi M, Alsaiari AA, Allahyani M, Aljuaid A, Salih M, Alghamdi A, Alrofaidi MA, Abida, Imran M (2023) Trofinetide for Rett syndrome: highlights on the development and related inventions of the first USFDA-approved treatment for rare pediatric unmet medical need. J Clin Med 12. 10.3390/jcm12155114 10.3390/jcm12155114 PMC10420089 37568516

[CR73] Jaffe GJ, Westby K, Csaky KG, Monés J, Pearlman JA, Patel SS, Joondeph BC, Randolph J, Masonson H, Rezaei KA (2021). C5 inhibitor avacincaptad pegol for geographic atrophy due to age-related macular degeneration: a randomized pivotal phase 2/3 trial. Ophthalmology.

[CR74] Jang J-H, Weyne J, Chaudhari U, Harari O, Miller J, Dain B, Meagher KA, Rodgers ML, Perlee L, Morton L, Weinreich DM, Yancopoulos GD, Griffin M (2021). Pozelimab, a human monoclonal antibody against complement factor C5, provided inhibition of intravascular hemolysis in patients with paroxysmal nocturnal hemoglobinuria. Blood.

[CR75] Jang JH, Wong L, Ko BS, Yoon SS, Li K, Baltcheva I, Nidamarthy PK, Chawla R, Junge G, Yap ES (2022). Iptacopan monotherapy in patients with paroxysmal nocturnal hemoglobinuria: a 2-cohort open-label proof-of-concept study. Blood Adv.

[CR76] Jenkins SW, Robinson DS, Fabre LF, Andary JJ, Messina ME, Reich LA (1990). Gepirone in the treatment of major depression. J Clin Psychopharmacol.

[CR77] Jin J, Zhong XB (2023). ASO drug Qalsody (tofersen) targets amyotrophic lateral sclerosis. Trends Pharmacol Sci.

[CR78] Johansen KL, Cobitz AR, Singh AK, Macdougall IC, Lopes RD, Obrador GT, Kovesdy CP, Israni R, Jha V, Okoro T, Sprys M, Jolly S, Lindsay AC, Bhatt P, Camejo RR, Keeley T, Cizman B, Wheeler DC (2023). The ASCEND-NHQ randomized trial found positive effects of daprodustat on hemoglobin and quality of life in patients with non-dialysis chronic kidney disease. Kidney Int.

[CR79] Johnson KA, Martin N, Nappi RE, Neal-Perry G, Shapiro M, Stute P, Thurston RC, Wolfman W, English M, Franklin C, Lee M, Santoro N (2023). Efficacy and safety of fezolinetant in moderate to severe vasomotor symptoms associated with menopause: a phase 3 RCT. J Clin Endocrinol Metab.

[CR80] Kang C (2023). Retifanlimab: first approval. Drugs.

[CR81] Kanter J, Walters MC, Krishnamurti L, Mapara MY, Kwiatkowski JL, Rifkin-Zenenberg S, Aygun B, Kasow KA, Pierciey FJ, Bonner M, Miller A, Zhang X, Lynch J, Kim D, Ribeil JA, Asmal M, Goyal S, Thompson AA, Tisdale JF (2022). Biologic and clinical efficacy of LentiGlobin for sickle cell disease. N Engl J Med.

[CR82] Kaplon H, Crescioli S, Chenoweth A, Visweswaraiah J, Reichert JM (2023). Antibodies to watch in 2023. Mabs.

[CR83] Kaye KS, Shorr AF, Wunderink RG, Du B, Poirier GE, Rana K, Miller A, Lewis D, O'Donnell J, Chen L, Reinhart H, Srinivasan S, Isaacs R, Altarac D (2023). Efficacy and safety of sulbactam-durlobactam versus colistin for the treatment of patients with serious infections caused by Acinetobacter baumannii-calcoaceticus complex: a multicentre, randomised, active-controlled, phase 3, non-inferiority clinical trial (ATTACK). Lancet Infect Dis.

[CR84] Kayki-Mutlu G, Michel MC (2021). A year in pharmacology: new drugs approved by the US Food and Drug Administration in 2020. Naunyn-Schmiedeberg's Arch Pharmacol.

[CR85] Kayki-Mutlu G, Aksoyalp ZS, Wojnowski L, Michel MC (2022). A year in pharmacology: new drugs approved by the US Food and Drug Administration in 2021. Naunyn Schmiedebergs Arch Pharmacol.

[CR86] Kayki-Mutlu G, Aksoyalp ZS, Wojnowski L, Michel MC (2023). A year in pharmacology: new drugs approved by the US Food and Drug Administration in 2022. Naunyn Schmiedebergs Arch Pharmacol.

[CR87] Kayser S, Levis MJ (2023). The clinical impact of the molecular landscape of acute myeloid leukemia. Haematologica.

[CR88] Keam SJ (2019). Toripalimab: first global approval. Drugs.

[CR89] Keam SJ (2023). Gepirone extended-release: first approval. Drugs.

[CR90] Keam SJ (2023). Momelotinib: first approval. Drugs.

[CR91] Keam SJ (2023). Pirtobrutinib: first approval. Drugs.

[CR92] Keam SJ (2023). Talquetamab: first approval. Drugs.

[CR93] Keam SJ (2023). Trofinetide: first approval. Drugs.

[CR94] Keam SJ (2023). Vamorolone: first approval. Drugs.

[CR95] Kern JS, Sprecher E, Fernandez MF, Schauer F, Bodemer C, Cunningham T, Löwe S, Davis C, Sumeray M, Bruckner AL, Murrell DF (2023). Efficacy and safety of Oleogel-S10 (birch triterpenes) for epidermolysis bullosa: results from the phase III randomized double-blind phase of the EASE study. Br J Dermatol.

[CR96] Killock D (2023). Newly diagnosed AML: quizartinib improves OS. Nat Rev Clin Oncol.

[CR97] Kim HR, Lim SM, Kim HJ, Hwang SK, Park JK, Shin E, Bae MK, Ou SH, Wang J, Jewell SS, Kang DR, Soo RA, Haack H, Kim JH, Shim HS, Cho BC (2013). The frequency and impact of ROS1 rearrangement on clinical outcomes in never smokers with lung adenocarcinoma. Ann Oncol.

[CR98] King B, Zhang X, Harcha WG, Szepietowski JC, Shapiro J, Lynde C, Mesinkovska NA, Zwillich SH, Napatalung L, Wajsbrot D, Fayyad R, Freyman A, Mitra D, Purohit V, Sinclair R, Wolk R (2023). Efficacy and safety of ritlecitinib in adults and adolescents with alopecia areata: a randomised, double-blind, multicentre, phase 2b–3 trial. Lancet.

[CR99] Kingwell K (2023). First CRISPR therapy seeks landmark approval. Nat Rev Drug Discov.

[CR100] Koury MJ, Haase VH (2015). Anaemia in kidney disease: harnessing hypoxia responses for therapy. Nat Rev Nephrol.

[CR101] Ladikou EE, Chevassut T, Pepper CJ, Pepper AG (2020). Dissecting the role of the CXCL12/CXCR4 axis in acute myeloid leukaemia. Br J Haematol.

[CR102] Larik MO, Iftekhar MA, Syed BU, Ansari O, Ansari M (2023). Nasal spray (Zavegepant) for migraines: a mini-review. Ann Med Surg (lond).

[CR103] Lavacchi D, Roviello G, Guidolin A, Romano S, Venturini J, Caliman E, Vannini A, Giommoni E, Pellegrini E, Brugia M, Pillozzi S, Antonuzzo L (2023) Evaluation of fruquintinib in the continuum of care of patients with colorectal cancer. Int J Mol Sci 24. 10.3390/ijms24065840 10.3390/ijms24065840 PMC10051170 36982913

[CR104] Lederman S, Ottery FD, Cano A, Santoro N, Shapiro M, Stute P, Thurston RC, English M, Franklin C, Lee M, Neal-Perry G (2023). Fezolinetant for treatment of moderate-to-severe vasomotor symptoms associated with menopause (SKYLIGHT 1): a phase 3 randomised controlled study. Lancet.

[CR105] Lee A (2023). Omaveloxolone: first approval. Drugs.

[CR106] Lesokhin AM, Tomasson MH, Arnulf B, Bahlis NJ, Miles Prince H, Niesvizky R, Rodriotaguez-Otero P, Martinez-Lopez J, Koehne G, Touzeau C, Jethava Y, Quach H, Depaus J, Yokoyama H, Gabayan AE, Stevens DA, Nooka AK, Manier S, Raje N, Iida S, Raab MS, Searle E, Leip E, Sullivan ST, Conte U, Elmeliegy M, Czibere A, Viqueira A, Mohty M (2023). Elranatamab in relapsed or refractory multiple myeloma: phase 2 MagnetisMM-3 trial results. Nat Med.

[CR107] Lin C, Schwarzbach A, Sanz J, Montesinos P, Stiff P, Parikh S, Brunstein C, Cutler C, Lindemans CA, Hanna R, Koh LP, Jagasia MH, Valcarcel D, Maziarz RT, Keating AK, Hwang WYK, Rezvani AR, Karras NA, Fernandes JF, Rocha V, Badell I, Ram R, Schiller GJ, Volodin L, Walters MC, Hamerschlak N, Cilloni D, Frankfurt O, McGuirk JP, Kurtzberg J, Sanz G, Simantov R, Horwitz ME (2023). Multicenter long-term follow-up of allogeneic hematopoietic cell transplantation with omidubicel: a pooled analysis of five prospective clinical trials. Transplant Cell Ther.

[CR108] Linhart A, Dostálová G, Nicholls K, West ML, Tøndel C, Jovanovic A, Giraldo P, Vujkovac B, Geberhiwot T, Brill-Almon E, Alon S, Chertkoff R, Rocco R, Hughes D (2023). Safety and efficacy of pegunigalsidase alfa in patients with Fabry disease who were previously treated with agalsidase alfa: results from BRIDGE, a phase 3 open-label study. Orphanet J Rare Dis.

[CR109] Lipton RB, Croop R, Stock DA, Madonia J, Forshaw M, Lovegren M, Mosher L, Coric V, Goadsby PJ (2023). Safety, tolerability, and efficacy of zavegepant 10 mg nasal spray for the acute treatment of migraine in the USA: a phase 3, double-blind, randomised, placebo-controlled multicentre trial. Lancet Neurol.

[CR110] Liu A, Zhao J, Shah M, Migliorati JM, Tawfik SM, Bahal R, Rasmussen TP, Manautou JE, Zhong XB (2022). Nedosiran, a candidate siRNA drug for the treatment of primary hyperoxaluria: design, development, and clinical studies. ACS Pharmacol Transl Sci.

[CR111] Lloyd MR, Wander SA, Hamilton E, Razavi P, Bardia A (2022). Next-generation selective estrogen receptor degraders and other novel endocrine therapies for management of metastatic hormone receptor-positive breast cancer: current and emerging role. Ther Adv Med Oncol.

[CR112] Mah JK, Clemens PR, Guglieri M, Smith EC, Finkel RS, Tulinius M, Nevo Y, Ryan MM, Webster R, Castro D, Kuntz NL, McDonald CM, Damsker JM, Schwartz BD, Mengle-Gaw LJ, Jackowski S, Stimpson G, Ridout DA, Ayyar-Gupta V, Baranello G, Manzur AY, Muntoni F, Gordish-Dressman H, Leinonen M, Ward LM, Hoffman EP, Dang UJ (2022). Efficacy and safety of vamorolone in Duchenne muscular dystrophy: a 30-month nonrandomized controlled open-label extension trial. JAMA Netw Open.

[CR113] Markham A, Keam SJ (2019). Sotagliflozin: first global approval. Drugs.

[CR114] Mato AR, Shah NN, Jurczak W, Cheah CY, Pagel JM, Woyach JA, Fakhri B, Eyre TA, Lamanna N, Patel MR, Alencar A, Lech-Maranda E, Wierda WG, Coombs CC, Gerson JN, Ghia P, Le Gouill S, Lewis DJ, Sundaram S, Cohen JB, Flinn IW, Tam CS, Barve MA, Kuss B, Taylor J, Abdel-Wahab O, Schuster SJ, Palomba ML, Lewis KL, Roeker LE, Davids MS, Tan XN, Fenske TS, Wallin J, Tsai DE, Ku NC, Zhu E, Chen J, Yin M, Nair B, Ebata K, Marella N, Brown JR, Wang M (2021). Pirtobrutinib in relapsed or refractory B-cell malignancies (BRUIN): a phase 1/2 study. Lancet.

[CR115] Mead RJ, Shan N, Reiser HJ, Marshall F, Shaw PJ (2023). Amyotrophic lateral sclerosis: a neurodegenerative disorder poised for successful therapeutic translation. Nat Rev Drug Discov.

[CR116] Mendell JR, Shieh PB, McDonald CM, Sahenk Z, Lehman KJ, Lowes LP, Reash NF, Iammarino MA, Alfano LN, Sabo B, Woods JD, Skura CL, Mao HC, Staudt LA, Griffin DA, Lewis S, Wang S, Potter RA, Singh T, Rodino-Klapac LR (2023). Expression of SRP-9001 dystrophin and stabilization of motor function up to 2 years post-treatment with delandistrogene moxeparvovec gene therapy in individuals with Duchenne muscular dystrophy. Front Cell Dev Biol.

[CR117] Menon D, Bril V (2022). Pharmacotherapy of generalized myasthenia gravis with special emphasis on newer biologicals. Drugs.

[CR118] Messersmith WA, Shapiro GI, Cleary JM, Jimeno A, Dasari A, Huang B, Shaik MN, Cesari R, Zheng X, Reynolds JM, English PA, McLachlan KR, Kern KA, LoRusso PM (2015). A phase I, dose-finding study in patients with advanced solid malignancies of the oral gamma-secretase inhibitor PF-03084014. Clin Cancer Res.

[CR119] Minson A, Dickinson M (2021). Glofitamab CD20-TCB bispecific antibody. Leuk Lymphoma.

[CR120] Mullard A (2023). FDA approves first-in-class AKT inhibitor. Nat Rev Drug Discov.

[CR121] Naeem A, Utro F, Wang Q, Cha J, Vihinen M, Martindale S, Zhou Y, Ren Y, Tyekucheva S, Kim AS, Fernandes SM, Saksena G, Rhrissorrakrai K, Levovitz C, Danysh BP, Slowik K, Jacobs RA, Davids MS, Lederer JA, Zain R, Smith CIE, Leshchiner I, Parida L, Getz G, Brown JR (2023). Pirtobrutinib targets BTK C481S in ibrutinib-resistant CLL but second-site BTK mutations lead to resistance. Blood Adv.

[CR122] Naqvi K, Ravandi F (2019). FLT3 inhibitor quizartinib (AC220). Leuk Lymphoma.

[CR123] Nisar A, Ahmed Z, Yuan H (2023) Novel therapeutic targets for migraine. Biomedicines 11. 10.3390/biomedicines11020569 10.3390/biomedicines11020569 PMC9952984 36831105

[CR124] Ozen A, Comrie WA, Ardy RC, Domínguez Conde C, Dalgic B, Beser ÖF, Morawski AR, Karakoc-Aydiner E, Tutar E, Baris S, Ozcay F, Serwas NK, Zhang Y, Matthews HF, Pittaluga S, Folio LR, Unlusoy Aksu A, McElwee JJ, Krolo A, Kiykim A, Baris Z, Gulsan M, Ogulur I, Snapper SB, Houwen RHJ, Leavis HL, Ertem D, Kain R, Sari S, Erkan T, Su HC, Boztug K, Lenardo MJ (2017). CD55 deficiency, early-onset protein-losing enteropathy, and thrombosis. N Engl J Med.

[CR125] Packer M (2023). Sotagliflozin for heart failure: what we know about trials and mechanisms. J Card Fail.

[CR126] Parent H, Ferranti A, Niswender C (2023). Trofinetide: a pioneering treatment for Rett syndrome. Trends Pharmacol Sci.

[CR127] Passamonti F, Mora B (2023). Myelofibrosis. Blood.

[CR128] Patel SS, Lally DR, Hsu J, Wykoff CC, Eichenbaum D, Heier JS, Jaffe GJ, Westby K, Desai D, Zhu L, Khanani AM (2023). Avacincaptad pegol for geographic atrophy secondary to age-related macular degeneration: 18-month findings from the GATHER1 trial. Eye (Lond).

[CR129] Pignolo RJ, Hsiao EC, Al Mukaddam M, Baujat G, Berglund SK, Brown MA, Cheung AM, De Cunto C, Delai P, Haga N, Kannu P, Keen R, Le Quan Sang KH, Mancilla EE, Marino R, Strahs A, Kaplan FS (2023). Reduction of new heterotopic ossification (HO) in the open-label, phase 3 MOVE trial of palovarotene for fibrodysplasia ossificans progressiva (FOP). J Bone Miner Res.

[CR130] Pitt B, Bhatt DL, Szarek M, Cannon CP, Leiter LA, McGuire DK, Lewis JB, Riddle MC, Voors AA, Metra M, Lund LH, Komajda M, Testani JM, Wilcox CS, Ponikowski P, Lopes RD, Ezekowitz JA, Sun F, Davies MJ, Verma S, Kosiborod MN, Steg PG (2023). Effect of sotagliflozin on early mortality and heart failure-related events: a post hoc analysis of SOLOIST-WHF. JACC Heart Fail.

[CR131] Qi M, Kinzer K, Danielson KK, Martellotto J, Barbaro B, Wang Y, Bui JT, Gaba RC, Knuttinen G, Garcia-Roca R, Tzvetanov I, Heitman A, Davis M, McGarrigle JJ, Benedetti E, Oberholzer J (2014). Five-year follow-up of patients with type 1 diabetes transplanted with allogeneic islets: the UIC experience. Acta Diabetol.

[CR132] Rais T, Khan A, Riaz R (2023). Elrexfio (elranatamab-bcmm): the game-changer in treatment of multiple myeloma. Rare Tumors.

[CR133] Rao VK, Webster S, Šedivá A, Plebani A, Schuetz C, Shcherbina A, Conlon N, Coulter T, Dalm VA, Trizzino A, Zharankova Y, Kulm E, Körholz J, Lougaris V, Rodina Y, Radford K, Bradt J, Kucher K, Relan A, Holland SM, Lenardo MJ, Uzel G (2023). A randomized, placebo-controlled phase 3 trial of the PI3Kδ inhibitor leniolisib for activated PI3Kδ syndrome. Blood.

[CR134] Reis J, Vender R, Torres T (2019). Bimekizumab: the first dual inhibitor of interleukin (IL)-17A and IL-17F for the treatment of psoriatic disease and ankylosing spondylitis. BioDrugs.

[CR135] Riaz R, Khan A, Siddiqui T (2023). Epcoritamab-bysp (Epkinly) - a phenomenal breakthrough in the treatment of diffuse large B-cell lymphoma. Rare Tumors.

[CR136] Ritchlin CT, Coates LC, McInnes IB, Mease PJ, Merola JF, Tanaka Y, Asahina A, Gossec L, Gottlieb AB, Warren RB, Ink B, Bajracharya R, Shende V, Coarse J, Landewé RB (2023). Bimekizumab treatment in biologic DMARD-naïve patients with active psoriatic arthritis: 52-week efficacy and safety results from the phase III, randomised, placebo-controlled, active reference BE OPTIMAL study. Ann Rheum Dis.

[CR137] Rohail S, Fareed A, Taimuri MA, Adnan A (2023). Nirogacestat and its potential impact on desmoid tumor. Rare Tumors.

[CR138] Rovin BH, Barratt J, Heerspink HJL, Alpers CE, Bieler S, Chae DW, Diva UA, Floege J, Gesualdo L, Inrig JK, Kohan DE, Komers R, Kooienga LA, Lafayette R, Maes B, Małecki R, Mercer A, Noronha IL, Oh SW, Peh CA, Praga M, Preciado P, Radhakrishnan J, Rheault MN, Rote WE, Tang SCW, Tesar V, Trachtman H, Trimarchi H, Tumlin JA, Wong MG, Perkovic V (2023). Efficacy and safety of sparsentan versus irbesartan in patients with IgA nephropathy (PROTECT): 2-year results from a randomised, active-controlled, phase 3 trial. Lancet.

[CR139] Saiyin T, Kirkham AM, Bailey AJM, Shorr R, Pineault N, Maganti HB, Allan DS (2023). Clinical outcomes of umbilical cord blood transplantation using ex vivo expansion: a systematic review and meta-analysis of controlled studies. Transplant Cell Ther.

[CR140] Sandborn WJ, Vermeire S, Peyrin-Biroulet L, Dubinsky MC, Panes J, Yarur A, Ritter T, Baert F, Schreiber S, Sloan S, Cataldi F, Shan K, Rabbat CJ, Chiorean M, Wolf DC, Sands BE, D'Haens G, Danese S, Goetsch M, Feagan BG (2023). Etrasimod as induction and maintenance therapy for ulcerative colitis (ELEVATE): two randomised, double-blind, placebo-controlled, phase 3 studies. Lancet.

[CR141] Sanghani NS, Haase VH (2019). Hypoxia-inducible factor activators in renal anemia: current clinical experience. Adv Chronic Kidney Dis.

[CR142] Schiffmann R, Goker-Alpan O, Holida M, Giraldo P, Barisoni L, Colvin RB, Jennette CJ, Maegawa G, Boyadjiev SA, Gonzalez D, Nicholls K, Tuffaha A, Atta MG, Rup B, Charney MR, Paz A, Szlaifer M, Alon S, Brill-Almon E, Chertkoff R, Hughes D (2019). Pegunigalsidase alfa, a novel PEGylated enzyme replacement therapy for Fabry disease, provides sustained plasma concentrations and favorable pharmacodynamics: a 1-year phase 1/2 clinical trial. J Inherit Metab Dis.

[CR143] Schoser B, Roberts M, Byrne BJ, Sitaraman S, Jiang H, Laforêt P, Toscano A, Castelli J, Díaz-Manera J, Goldman M, van der Ploeg AT, Bratkovic D, Kuchipudi S, Mozaffar T, Kishnani PS (2021). Safety and efficacy of cipaglucosidase alfa plus miglustat versus alglucosidase alfa plus placebo in late-onset Pompe disease (PROPEL): an international, randomised, double-blind, parallel-group, phase 3 trial. Lancet Neurol.

[CR144] Sheppard JD, Kurata F, Epitropoulos AT, Krösser S, Vittitow JL (2023). NOV03 for signs and symptoms of dry eye disease associated with meibomian gland dysfunction: the randomized phase 3 MOJAVE study. Am J Ophthalmol.

[CR145] Shirley M (2018). Fruquintinib: first global approval. Drugs.

[CR146] Shirley M (2023). Glofitamab: first approval. Drugs.

[CR147] Shirley M (2023). Zilucoplan: first approval. Drugs.

[CR148] Silverberg JI, Bissonnette R, Kircik L, Murrell DF, Selfridge A, Liu K, Ahluwalia G, Guttman-Yassky E (2023). Efficacy and safety of etrasimod, a sphingosine 1-phosphate receptor modulator, in adults with moderate-to-severe atopic dermatitis (ADVISE). J Eur Acad Dermatol Venereol.

[CR149] Smith B, Kiessling A, Lledo-Garcia R, Dixon KL, Christodoulou L, Catley MC, Atherfold P, D'Hooghe LE, Finney H, Greenslade K, Hailu H, Kevorkian L, Lightwood D, Meier C, Munro R, Qureshi O, Sarkar K, Shaw SP, Tewari R, Turner A, Tyson K, West S, Shaw S, Brennan FR (2018). Generation and characterization of a high affinity anti-human FcRn antibody, rozanolixizumab, and the effects of different molecular formats on the reduction of plasma IgG concentration. MAbs.

[CR150] Strober B, Paul C, Blauvelt A, Thaçi D, Puig L, Lebwohl M, White K, Vanvoorden V, Deherder D, Gomez NN, Eyerich K (2023). Bimekizumab efficacy and safety in patients with moderate to severe plaque psoriasis: two-year interim results from the open-label extension of the randomized BE RADIANT phase 3b trial. J Am Acad Dermatol.

[CR151] Surasi DS, Eiber M, Maurer T, Preston MA, Helfand BT, Josephson D, Tewari AK, Somford DM, Rais-Bahrami S, Koontz BF, Bostrom PJ, Chau A, Davis P, Schuster DM, Chapin BF (2023). Diagnostic performance and safety of positron emission tomography with (18)F-rhPSMA-7.3 in patients with newly diagnosed unfavourable intermediate- to very-high-risk prostate cancer: results from a phase 3, prospective, multicentre study (LIGHTHOUSE). Eur Urol.

[CR152] Tang GQ, Tang Y, Dhamnaskar K, Hoarty MD, Vyasamneni R, Vadysirisack DD, Ma Z, Zhu N, Wang JG, Bu C, Cong B, Palmer E, Duda PW, Sayegh C, Ricardo A (2023). Zilucoplan, a macrocyclic peptide inhibitor of human complement component 5, uses a dual mode of action to prevent terminal complement pathway activation. Front Immunol.

[CR153] Tauber J, Berdy GJ, Wirta DL, Krösser S, Vittitow JL (2023). NOV03 for dry eye disease associated with meibomian gland dysfunction: results of the randomized phase 3 GOBI study. Ophthalmology.

[CR154] Thompson GR, Soriano A, Skoutelis A, Vazquez JA, Honore PM, Horcajada JP, Spapen H, Bassetti M, Ostrosky-Zeichner L, Das AF, Viani RM, Sandison T, Pappas PG (2021). Rezafungin versus caspofungin in a phase 2, randomized, double-blind study for the treatment of candidemia and invasive candidiasis: the STRIVE trial. Clin Infect Dis.

[CR155] Thompson GR, Soriano A, Cornely OA, Kullberg BJ, Kollef M, Vazquez J, Honore PM, Bassetti M, Pullman J, Chayakulkeeree M, Poromanski I, Dignani C, Das AF, Sandison T, Pappas PG (2023). Rezafungin versus caspofungin for treatment of candidaemia and invasive candidiasis (ReSTORE): a multicentre, double-blind, double-dummy, randomised phase 3 trial. Lancet.

[CR156] Tremblay D, Mesa R (2022). Momelotinib for the treatment of myelofibrosis with anemia. Future Oncol.

[CR157] U.S. Food and Drug Administration (2023a) https://www.fda.gov/vaccines-blood-biologics/cellular-gene-therapy-products/approved-cellular-and-gene-therapy-products Accessed 23.12.2023

[CR158] U.S. Food and Drug Administration (2023b) https://www.fda.gov/drugs/new-drugs-fda-cders-new-molecular-entities-and-new-therapeutic-biological-products/novel-drug-approvals-2023. Accessed 29 Jan 2024

[CR159] Verkleij CPM, Broekmans MEC, van Duin M, Frerichs KA, Kuiper R, de Jonge AV, Kaiser M, Morgan G, Axel A, Boominathan R, Sendecki J, Wong A, Verona RI, Sonneveld P, Zweegman S, Adams HC, Mutis T, van de Donk N (2021). Preclinical activity and determinants of response of the GPRC5DxCD3 bispecific antibody talquetamab in multiple myeloma. Blood Adv.

[CR160] Verstovsek S, Mesa R, Gupta V, Lavie D, Dubruille V, Cambier N, Platzbecker U, Hus M, Xicoy B, Oh ST, Kiladjian JJ, Vannucchi AM, Gerds A, Egyed M, Mayer J, Sacha T, Kawashima J, Morris M, Huang M, Harrison C (2023). Momelotinib long-term safety and survival in myelofibrosis: integrated analysis of phase 3 randomized controlled trials. Blood Adv.

[CR161] Watkins RR, Du B, Isaacs R, Altarac D (2023). Pathogen-targeted clinical development to address unmet medical need: design, safety, and efficacy of the ATTACK trial. Clin Infect Dis.

[CR162] Yadollahikhales G, Rojas JC (2023). Anti-amyloid immunotherapies for Alzheimer’s disease: a 2023 clinical update. Neurotherapeutics.

[CR163] Yeu E, Wirta DL, Karpecki P, Baba SN, Holdbrook M (2023). Lotilaner ophthalmic solution, 0.25%, for the treatment of Demodex blepharitis: results of a prospective, randomized, vehicle-controlled, double-masked, pivotal trial (saturn-1). Cornea.

[CR164] Yun MR, Kim DH, Kim SY, Joo HS, Lee YW, Choi HM, Park CW, Heo SG, Kang HN, Lee SS, Schoenfeld AJ, Drilon A, Kang SG, Shim HS, Hong MH, Cui JJ, Kim HR, Cho BC (2020). Repotrectinib exhibits potent antitumor activity in treatment-naive and solvent-front-mutant ROS1-rearranged non-small cell lung cancer. Clin Cancer Res.

[CR165] Zadik Z, Zelinska N, Iotova V, Skorodok Y, Malievsky O, Mauras N, Valluri SR, Pastrak A, Rosenfeld R (2023). An open-label extension of a phase 2 dose-finding study of once-weekly somatrogon vs. once-daily Genotropin in children with short stature due to growth hormone deficiency: results following 5 years of treatment. J Pediatr Endocrinol Metab.

[CR166] Zaidman CM, Proud CM, McDonald CM, Lehman KJ, Goedeker NL, Mason S, Murphy AP, Guridi M, Wang S, Reid C, Darton E, Wandel C, Lewis S, Malhotra J, Griffin DA, Potter RA, Rodino-Klapac LR, Mendell JR (2023). Delandistrogene moxeparvovec gene therapy in ambulatory patients (aged ≥4 to <8 years) with Duchenne muscular dystrophy: 1-year interim results from study SRP-9001-103 (ENDEAVOR). Ann Neurol.

[CR167] Zhang L, Hao B, Geng Z, Geng Q (2021). Toripalimab: the first domestic anti-tumor PD-1 antibody in China. Front Immunol.

[CR168] Zheng C, Huang J, Xu G, Li W, Weng X, Zhang S (2024). The Notch signaling pathway in desmoid tumor: recent advances and the therapeutic prospects. Biochim Biophys Acta Mol Basis Dis.

